# Burn‐Safe Biodegradable Magnetocaloric Composites for Temperature‐Controlled Biomedical Applications

**DOI:** 10.1002/advs.202509914

**Published:** 2025-11-25

**Authors:** Pornpawee Uliss, Vasiliki Gkouzioti, Wuliji Hanggai, Friso Kahler, Elena Aprea, Qi Jia, Jean‐Philippe Frimat, Ekkes Brück, Clementine M. Boutry

**Affiliations:** ^1^ Faculty of Electrical Engineering Mathematics and Computer Science (EEMCS) Department of Microelectronics (ME), Electronic Components, Technology and Materials (ECTM) Delft University of Technology Delft 2628 CD The Netherlands; ^2^ Department of Neurology and Department of Human Genetics Leiden University Medical Center Leiden 2333 ZC The Netherlands; ^3^ Faculty of Applied Sciences Department of Radiation Science and Technology (RST) Fundamental Aspects of Materials and Energy (FAME) Delft University of Technology Delft 2628 CD The Netherlands; ^4^ Faculty of Applied Sciences Department of Radiation Science and Technology (RST) Applied Radiation and Isotopes (ARI) Delft University of Technology Delft 2628 CD The Netherlands

**Keywords:** biodegradable composites, biomedical applications, burn‐safe composites, magnetocaloric materials, magnetothermal therapy, temperature‐responsive particles, tunable Curie temperature

## Abstract

Magnetothermal stimulation is key in biomedical applications like tumor ablation, drug delivery, and regenerative therapies. A common method involves injecting magnetic particles that heat under an alternating magnetic field (AMF). However, uncontrolled heating can damage healthy tissues. Maintaining temperatures below 45 °C is critical. Using materials with a Curie temperature (*T_c_*) near this limit offers a self‐regulating solution, as magnetization—and thus heating—drops sharply at *T_c_*. This study explores Mn_0.65_Fe_1.30_P_0.65_Si_0.37_ (MCM), a magnetocaloric material composed of non‐toxic elements and featuring a tunable *T_c_*. It is engineered to exhibit a *T_c_* of 43 °C, close to the safe physiological threshold. MCM particles are encapsulated in a wax matrix to form a composite that responds to AMF exposure. Heat generated by MCM particles triggers the wax phase transition, while the obtained *T_c_* enables the composite to achieve self‐limiting thermal regulation under magnetic field exposure. Biocompatibility tests using human umbilical vein endothelial cells (HUVECs) show over 90% cell viability in direct and indirect contact. Stability tests in phosphate buffers at 37 °C confirm controlled degradation over 28 days. These results demonstrate that MCM is a promising, burn‐free magnetic material for safe, localized heating, supporting its use in self‐regulating, temperature‐responsive biomedical systems.

## Graphical abstract

This work investigates a magnetocaloric (Mn,Fe)_2_(P,Si) compound tailored for precise, self‐regulating heating in biomedical use. With a Curie temperature tuned at 43 °C, it enables therapeutic activation without overheating. Encapsulation in a composite ensures controlled temperature rise under an alternating magnetic field (AMF), with high biocompatibility and stability, hinting at a new frontier in safe, localized thermal medical treatments.

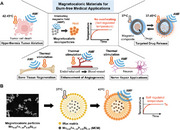

## 1 Introduction

**Figure 1:**
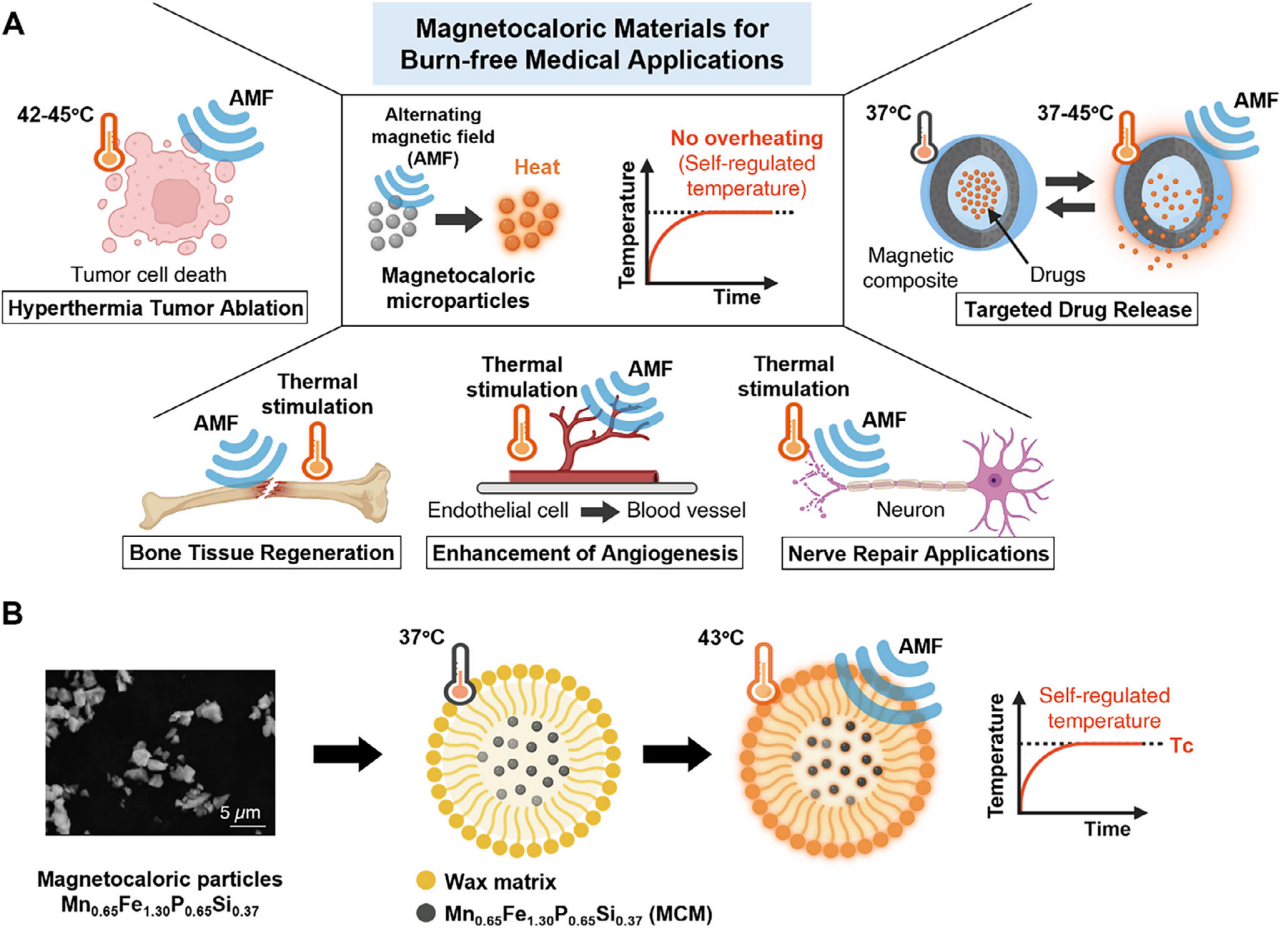
A) Schematic illustration depicting potential biomedical applications of burn‐free magnetocaloric materials, including hyperthermia‐based tumor ablation, targeted drug delivery, and regenerative medicine through thermally responsive stimulation for tissue and cell regeneration. B) Schematic representation of the magnetocaloric Mn_0.65_Fe_1.30_P_0.65_Si_0.37_ (MCM)‐wax composite system developed in this study. Created with BioRender.com.

Several medical applications demand precise and localized temperature control at specific sites within the body, particularly in hyperthermia‐based tumor ablation,^[^ ^1^, ^2^, ^3^, ^4^, ^5^ ^]^ targeted drug delivery,^[^ ^6^, ^7^, ^8^, ^9^, ^10^ ^]^ and regenerative medicine (**Figure** **1**A).^[^ ^11^, ^12^, ^13^ ^]^ A widely used strategy for generating localized heating involves the injection of functionalized particles that selectively accumulate at targeted tissue sites. These particles can be remotely activated by external stimuli, such as infrared light, which induces a photothermal response in gold nanoparticles,^[^ ^14^, ^15^ ^]^ or alternating magnetic fields (AMFs), which trigger a magnetothermal response in magnetic particles such as Fe_3_O_4_ and γFe_2_O_3_.^[^ ^1^, ^2^, ^16^, ^17^ ^]^ To date, magnetic agents that generate heat under alternating magnetic fields have attracted considerable attention owing to several distinct advantages, including deep tissue penetration without significant attenuation, remote controllability, and precise on‐demand activation.^[^ ^7^, ^18^ ^]^ Despite its potential, a primary challenge of heating induced by alternating magnetic fields lies in the absence of intrinsic thermal regulation at the targeted site. Upon exposure, magnetic particles generate heat through magnetothermal effects, which can result in temperature elevations exceeding safe physiological limits, thereby increasing the risk of damage to surrounding healthy tissues. Particularly, for therapeutic applications such as magnetic hyperthermia^[^ ^5^, ^19^, ^20^ ^]^ and regenerative medicine,^[^ ^13^, ^21^, ^22^, ^23^, ^24^ ^]^ maintaining localized temperature below the physiological safety threshold of 45 °C is critical to prevent unintended and potentially irreversible damage, including cellular apoptosis or necrosis.^[^ ^21^, ^25^, ^26^ ^]^ Current therapeutic interventions typically involve adjusting the magnetic field strength, frequency, or duration of AMF exposure to maintain temperatures within safe thermal thresholds. However, clinical translation remains challenging due to the inability to precisely control and monitor localized temperature in vivo during stimulation by alternating magnetic fields.^[^ ^27^ ^]^ Therefore, there is a pressing need for non‐invasive strategies capable of delivering precise thermal control, thereby minimizing both subtherapeutic effects and unintended thermal damage to surrounding healthy tissues.^[^ ^28^, ^29^ ^]^

A key strategy for thermally stimulated applications involves engineering magnetic particles with self‐regulating temperature mechanisms. This can be achieved by tuning their Curie temperature (*T_c_*) to correspond with the desired therapeutic safety threshold. Curie temperature is defined as the critical point at which a magnetic phase transition occurs. Below *T_c_*, ferri‐ or ferromagnetic particles generate heat through magnetothermal mechanisms, including hysteresis losses^[^ ^30^ ^]^ or relaxation losses.^[^ ^27^ ^]^ As the temperature approaches the Curie temperature, thermal agitation drives a transition from a magnetically ordered ferri‐ or ferromagnetic state to a disordered paramagnetic state, resulting in a rapid decline in magnetic losses, consequently, a reduction in heating efficiency. This self‐limiting effect effectively halts further temperature increase, even under continuous exposure to alternating magnetic fields. By exploiting the temperature‐dependent magnetic properties at the Curie temperature, it is possible to develop a heating system with an inherent thermal switch‐off mechanism, thereby mitigating the risk of overheating and eliminating the need for external temperature monitoring. However, the Curie temperature of most existing magnetic materials significantly exceeds the physiological safety range (below 45 °C), limiting their applicability in biomedical thermal therapies. For instance, Fe_3_O_4_ and γ‐Fe_2_O_3_ exhibit Curie temperatures of 585 and 447 °C, respectively,^[^ ^31^ ^]^ which are higher than the physiological safety threshold. Although doping magnetic particles with weakly magnetic or non‐magnetic elements can lower the Curie temperature,^[^ ^32^, ^33^ ^]^ this approach often compromises biocompatibility. Alternatively, Ni‐Pd alloys present more suitable Curie temperature values ranging from 43 to 58 °C,^[^ ^34^ ^]^ yet their high cost and complex synthesis processes pose significant challenges for scalable production.^[^ ^35^ ^]^ Therefore, developing effective strategies to precisely modulate the Curie temperature of magnetic particles within the physiologically safe range—while maintaining both biocompatibility, ease of synthesis, and sufficient heating efficiency—remains essential for the advancement of safe and clinically effective thermal therapies.

Magnetocaloric materials, particularly those exhibiting a giant magnetocaloric effect associated with a first‐order magnetic phase transition, show strong potential for self‐regulating thermal control. The abrupt change in magnetization near the Curie temperature, driven by strong magneto‐structural coupling, produces a sharp thermal response that can effectively moderate further temperature increases. This self‐limiting behavior reduces the reliance on precise external magnetic field control and supports non‐invasive, localized temperature management, both of which are highly advantageous for biomedical applications. While this self‐regulation is not absolute and remains influenced by the strength of the applied magnetic field, the inherent tendency of the material to stabilize temperature near its Curie point, especially when *T_c_* is tuned to a physiologically safe range, provides an additional layer of thermal safety when the magnetic field is properly optimized. Additionally, the pronounced ordered magnetic moments with the giant magnetocaloric effect enhance sensitivity to moderate alternating magnetic fields, thereby increasing energy efficiency relative to conventional magnetic materials, which typically require stronger fields to achieve comparable heating performance. Notably, first‐order magnetic transition materials are rare in nature but can be engineered to exhibit tunable Curie temperatures within the desired range by tailoring their structural or elastic transitions to coincide with the magnetic transition.^[^ ^36^ ^]^ Ongoing research seeks to tailor the Curie temperature of magnetocaloric materials to align with the safe physiological window. For instance, rare earth gadolinium (Gd) has been investigated. Ahmad et al. examined Gd_2_C and Gd_5_Si_4_ with Curie temperature in the therapeutic hyperthermia range of ≈40 to 60 °C.^[^ ^37^ ^]^ However, prolonged milling significantly alters the structural integrity of Gd particles, resulting in reduced magnetization and shifts in the Curie temperature.^[^ ^38^, ^39^ ^]^ Moreover, biocompatibility concerns arise from the toxicity of Gd^3+^ ions,^[^ ^40^ ^]^ along with its high cost, limits the scalability of gadolinium‐based materials for clinical applications.^[^ ^41^ ^]^ Conversely, Soleymani et al. tailored La_0.73_Sr_0.27_MnO_3_ to exhibit a tunable Curie temperature between 59 and 75 °C,^[^ ^31^ ^]^ although these values exceed the optimal range for intended thermal therapies. Therefore, the development of non‐toxic magnetocaloric materials with a tunable Curie temperature within the physiologically safe range—below 45 °C—holds significant promise for biomedical applications that require effective temperature control.

In this study, we investigate the magnetocaloric compound (Mn,Fe)_2_(P,Si) for biomedical thermal applications, due to its absence of toxic constituents, magnetocaloric effect with a sharp first‐order magnetic phase transition, and cost‐effectiveness.^[^ ^36^, ^42^ ^]^ Moreover, its Curie temperature can be easily tuned by adjusting the ratios of Mn, Fe, P, and Si,^[^ ^42^ ^]^ and it also exhibits structural and thermal stability, even after extended high‐energy milling from micro‐ to nanoscale, without significant drift in the Curie temperature.^[^ ^43^ ^]^ These attributes make (Mn,Fe)_2_(P,Si) a highly promising candidate for efficient, self‐regulating thermal actuation in biomedical settings. Nevertheless, current research remains primarily focused on materials optimization, such as T_c_ tuning, hysteresis reduction, and nanoparticle fabrication, while investigations addressing the key aspects required to translate this material into practical biomedical applications are still lacking. Here, we demonstrate the feasibility of fabricating a magnetocaloric wax composite capable of magnetically triggered, self‐controlled heating that induces wax melting within the physiologically safe range, as shown in Figure 1B. Wax is well‐suited for lipid‐based drug delivery vehicles due to its biocompatibility, high encapsulation efficiency, and ability to protect therapeutic agents from degradation and premature release.^[^ ^44^ ^]^ Magnetocaloric Mn_0.65_Fe_1.30_P_0.65_Si_0.37_ (MCM) particles are synthesized and compositionally tailored to exhibit a Curie temperature of 43 °C. A multi‐step process involving high‐energy ball milling, melt spinning, annealing, and a second surfactant‐assisted ball milling is employed to optimize the material's structural and magnetic properties. The lipidic matrix of the composite consists of a blend of bayberry and lanolin waxes incorporating synthesized MCM particles. The heating performance of the MCM‐wax composite is evaluated under alternating magnetic fields and compared with conventional particles such as Fe_3_O_4_ and carbonyl iron powder (CIP). Owing to the finely tuned Curie temperature, the MCM‐wax composite demonstrates effective temperature self‐regulation, acting as an intrinsic thermal switch‐off mechanism. Its heating performance is further validated under varying field amplitudes, and biocompatibility and degradability assessments further support its potential for future in vivo applications. Collectively, Mn_0.65_Fe_1.30_P_0.65_Si_0.37_ emerges as a biocompatible, self‐regulating magnetocaloric material capable of precise thermal control within the physiological safety window, highlighting its strong potential for integration into next‐generation thermoresponsive biomedical platforms.

## 2 Result and Discussion

### 2.1 Synthesis and Structural Characterization of Mn_0.65_Fe_1.30_P_0.65_Si_0.37_

**Figure 2:**
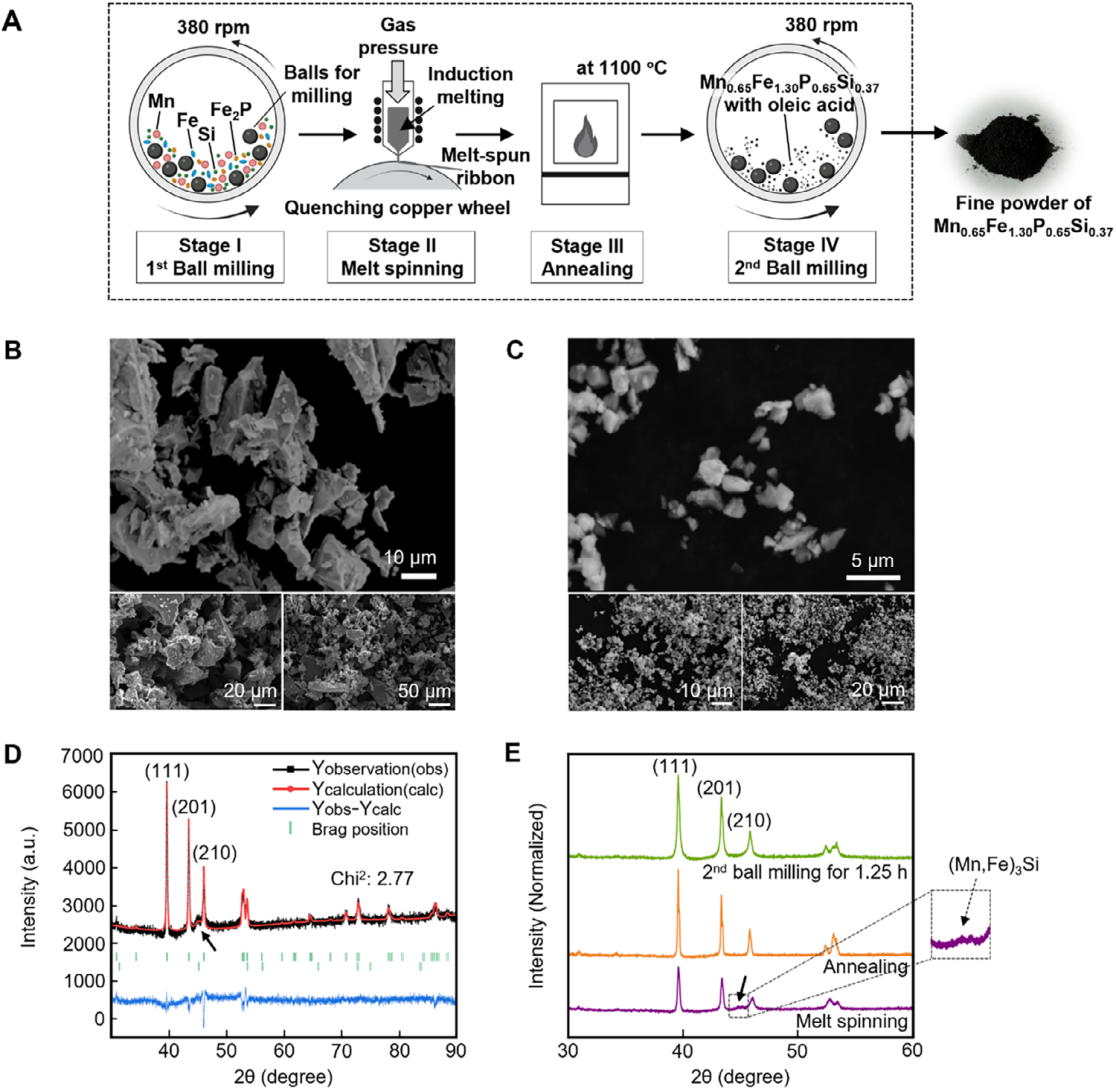
A) Schematic illustration of the preparation process for magnetocaloric Mn_0.65_Fe_1.30_P_0.65_Si_0.37_ particles. Created with BioRender.com. B) SEM images of Mn_0.65_Fe_1.30_P_0.65_Si_0.37_ polycrystalline particles obtained after the first ball milling (Stage I) for 10 h, followed by melt spinning (Stage II) and annealing (Stage III). C) SEM images of Mn_0.65_Fe_1.30_P_0.65_Si_0.37_ polycrystalline particles obtained after the second ball milling (Stage IV) for 1.25 h. D) XRD pattern of the bulk magnetocaloric Mn_0.65_Fe_1.30_P_0.65_Si_0.37_ particles after melt spinning (Stage II), showing the dominant Fe_2_P‐type crystal structure. The three main reflections—(111), (201), and (210)—are characteristic of this main phase. The black arrow indicates the presence of a secondary cubic (Mn,Fe)_3_Si impurity phase. E) XRD patterns of Mn_0.65_Fe_1.30_P_0.65_Si_0.37_ particles obtained from different synthesis procedures, all exhibiting the dominant Fe_2_P‐type crystal structure, as indicated by the characteristic (111), (201), and (210) reflections. The inset shows a magnified view of the region around the characteristic peak of the 3:1 (Mn,Fe)_3_Si phase.

In this study, Mn_0.65_Fe_1.30_P_0.65_Si_0.37_ (MCM) is synthesized using a laboratory‐scale alloy synthesis approach comprising four stages: high‐energy ball milling (HEBM, Stage I), melt spinning (Stage II), subsequent heat treatment (annealing, Stage III), and a second surfactant‐assisted ball milling (Stage IV). A schematic overview of the fabrication steps is presented in **Figures** **2**A and S1 (Supporting Information). Following the first milling step (Stage I), the powder is compacted into pellets and processed through melt spinning (Stage II). At this stage, the pellets are melted in a quartz tube using induction heating, and the resulting molten metal is expelled under gas pressure through a fine nozzle onto a rotating copper wheel, where it rapidly solidifies into a ribbon. This process facilitates the removal of oxide impurities.^[^ ^45^ ^]^ Moreover, implementing melt spinning prior to annealing offers a time‐efficient and less energy‐intensive alternative to conventional approaches such as double solid‐state sintering.^[^ ^46^, ^47^ ^]^ The particle size distribution of the Mn_0.65_Fe_1.30_P_0.65_Si_0.37_ powders is characterized by scanning electron microscopy (SEM), as shown in Figure 2B,C. During the first ball milling (Stage I), compositional homogeneity is achieved by subjecting the raw materials to repeated high‐energy collisions, which facilitate the rupture of chemical bonds, reduction in particle size, and generation of fresh reactive surfaces.^[^ ^48^ ^]^ As shown in Figure 2B, SEM images of Mn_0.65_Fe_1.30_P_0.65_Si_0.37_ (MCM) polycrystalline particles following the first ball milling (Stage I), melt spinning (Stage II), and subsequent annealing (Stage III) reveal a particle size distribution, ranging from ≈10 to 30 µm. This wide distribution and poor uniformity of the MCM particles are likely attributed to the absence of surfactants during synthesis. Surfactants typically inhibit the cold‐welding effect,^[^ ^49^ ^]^ a phenomenon in which particles adhere to one another and agglomerate under high‐energy collisions, leading to the formation of larger aggregates. To address this limitation and achieve a smaller, more uniform particle size distribution, a second milling step (Stage IV) is performed in the presence of a surfactant. The material is ball milled for 1.25 h (Stage IV) using 10 wt.% oleic acid (C_18_H_34_O_2_) as a surfactant. As shown in Figure 2C, SEM images of the MCM polycrystalline particles after the second ball milling with surfactant exhibit a marked improvement in particle morphology. The presence of surfactants aids in preserving the crystal structure of the magnetic phase and mitigates particle oxidation during and after the milling process.^[^ ^50^ ^]^ For each sample subjected to different second ball‐milling durations (1.25, 2.5, and 5 h), several hundred particles are randomly selected from the SEM images to determine the particle size distribution, including the average particle size and standard deviation (Figure S2, Supporting Information). The results indicate that the average particle size decreases progressively from ≈3 µm after 1.25 h of milling to 900 nm after 2.5 h, and further to 720 nm after 5 h. SEM images also show that there is a decrease in the particle size with extended milling, and no significant change in the particle morphology.

The X‐ray diffraction (XRD) pattern of the melt‐spun Mn_0.65_Fe_1.30_P_0.65_Si_0.37_ sample, as shown in Figure 2D, reveals a dominant hexagonal Fe_2_P‐type crystal structure (space group *P‐62m*), characterized by three strong reflections—(111), (201), and (210)—which are indicative of the primary phase. However, an impurity phase corresponding to cubic (Mn,Fe)_3_Si (space group *Fm‐3m*) is also detected, indicated by the black arrow in Figure 2D, with a quantified weight fraction of 31.76(2.81) wt.%. The presence of this impurity phase is attributed to microstructural inhomogeneity, which can adversely affect both the structural stability and magnetic performance of the material.^[^ ^51^ ^]^ To improve phase purity and microstructural uniformity, a short annealing treatment is applied following the melt‐spinning process.^[^ ^52^ ^]^ As illustrated in Figure 2E, the XRD pattern of the annealed sample displays dominant Fe_2_P‐type reflections, with the (111), (201), and (210) peaks clearly visible, confirming the preservation of the hexagonal crystal structure in the milled powders. Notably, no detectable peaks corresponding to the impurity phase of (Mn,Fe)_3_Si are observed in the annealed sample, as evidenced by the absence of intensity at the position of the strongest 3:1 impurity phase reflection, indicated by the inset and black arrows in Figure 2E. This indicates that annealing effectively eliminates impurity phases and significantly enhances the phase purity of the material.

As shown in Figure 2E, the XRD peaks exhibit broadening following the second ball milling process, suggesting a reduction in crystallite size. To further assess the influence of the second ball milling duration on crystallite size refinement, the MCM sample is re‐synthesized. The ribbon‐like sample obtained after annealing is subjected to the second milling step with 10 wt.% oleic acid for 1.25, 2.5, and 5 h. The observed progressive peak broadening, as well as a gradual reduction in XRD peak intensity (Figure S3, Supporting Information), present the direct impact of ball‐milling time on the size reduction. The average crystallite size of the MCM sample is then estimated using the Scherrer equation (Equation 1 in the Experimental Section). Although some strains may contribute to peak broadening, their effect is not considered in the calculation because: i) when crystallite sizes are below 100 nm, the peak broadening due to crystallite size typically dominates over that from microstrain, and ii) the (111) reflection at a low diffraction angle is selected for crystallite size estimation as the influence of microstrain on peak broadening is relatively small in this range.^[^ ^43^ ^]^ As shown in **Table** **1**, the average crystallite size increases from 45.5 ± 2.5 nm to 496.4 ± 189.4 nm after the annealing process, which is attributed to grain growth within the microstructure during the heat treatment process. Notably, the increase in grain size contributes to an enhancement in saturation magnetization.^[^ ^46^ ^]^ Subsequently, the crystallite size is significantly reduced to 29.9 ± 0.9 nm following the second ball‐milling for 1.25 h. A progressive decrease in crystallite size is also observed in the re‐synthesized sample with increasing ball milling duration, as shown in **Table** **2**. The crystallite size decreases from 39.7 ± 0.7 nm after 1.25 h to 31.1 ± 0.5 nm after 2.5 h, and further to 27.1 ± 0.4 nm after 5 h. This trend is consistent with the continuous decrease in particle size revealed by the SEM‐based particle size distribution analysis (Figure S2, Supporting Information). The size reduction is primarily attributed to intense plastic deformation and continuous fracturing caused by prolonged milling. As milling time increases, the repeated collisions between milling balls and particles promote progressive crystallite refinement.

**Table 1:** The average crystallite size of Mn_0.65_Fe_1.30_P_0.65_Si_0.37_ particles from different synthesis steps with their corresponding standard errors (±).

Synthesis step	Crystallite size [nm]
Melt spinning (Stage II)	45.5 ± 2.5
Annealing (Stage III)	496.4 ± 189.4
2nd ball milling (Stage IV) for 1.25 h	29.9 ± 0.9

**Table 2:** The average crystallite size of re‐synthesized Mn_0.65_Fe_1.30_P_0.65_Si_0.37_ samples following the second ball milling step, measured at various durations (in hours). Values are reported with their corresponding standard errors (±).

Synthesis step	Crystallite size [nm]
Annealing (Stage III)	484.4 ± 44.4
2nd ball milling (Stage IV) for 1.25 h	39.7 ± 0.7
2nd ball milling (Stage IV) for 2.5 h	31.1 ± 0.5
2nd ball milling (Stage IV) for 5 h	27.1 ± 0.4

Particle size significantly impacts biodistribution, physiological clearance, and magnetic behavior in biomedical systems. For localized administration platforms, such as thermally‐responsive drug release, magnetic hyperthermia, or micro‐electro‐mechanical (MEMS)‐based magnetic composites, microscale particles offer an effective balance between biological safety and functional performance. The magnetic response is highly dependent on particle dimension, which governs the shift from long‐range ferromagnetic ordering characteristic of micrometer‐sized particles to superparamagnetic behavior at the nanoscale. While superparamagnetic nanoparticles excel in systemic circulation by minimizing embolism risks and penetrating biological barriers for intracellular delivery,^[^ ^53^ ^]^ reduced particle size weakens long‐range magnetic ordering, lowering saturation magnetization and broadening the Curie temperature transition.^[^ ^43^, ^54^ ^]^ This effect impairs temperature control precision and compromises self‐regulated magnetothermal functionality. Conversely, microparticles often contain multiple magnetic domains, resulting in greater overall magnetization than single‐domain nanoparticles.^[^ ^55^ ^]^ The enhanced dipolar coupling among these domains also increases magnetic interactions, boosting heating efficiency under alternating magnetic fields. This strong magnetoresponsiveness makes microparticles ideal for magnetically actuated and thermally responsive biomedical devices like actuators, sensors, and hyperthermia agents. To ensure both biocompatibility and magnetic functionality, particles sized below ≈6 µm can traverse pulmonary capillaries (7–10 µm in diameter), whereas particles over 10 µm are typically retained in the lungs and may pose a risk of capillary blockage.^[^ ^56^ ^]^ Thus, selecting particles smaller than this threshold effectively reduces the risk of embolism while maintaining strong magnetothermal and actuation properties. Our findings confirm that surfactant‐assisted high‐energy ball milling enables progressive downsizing of (Mn,Fe)_2_(P,Si)‐based magnetocaloric particles from the microscale toward near‐nanoscale dimensions. While micron‐sized particles offer enhanced hysteresis losses and higher magnetization, their shape, tendency to cluster, and distribution within tissues must be carefully considered for each specific application. Microparticles often display less uniform dispersion in biological environments, and factors such as aggregation or irregular morphology can lead to non‐uniform heating and compromise temperature self‐regulation,^[^ ^57^ ^]^ both of which are critical for achieving safe and controlled biomedical magnetothermal therapy.

### 2.2 Magnetic Properties of Mn_0.65_Fe_1.30_P_0.65_Si_0.37_

**Figure 3:**
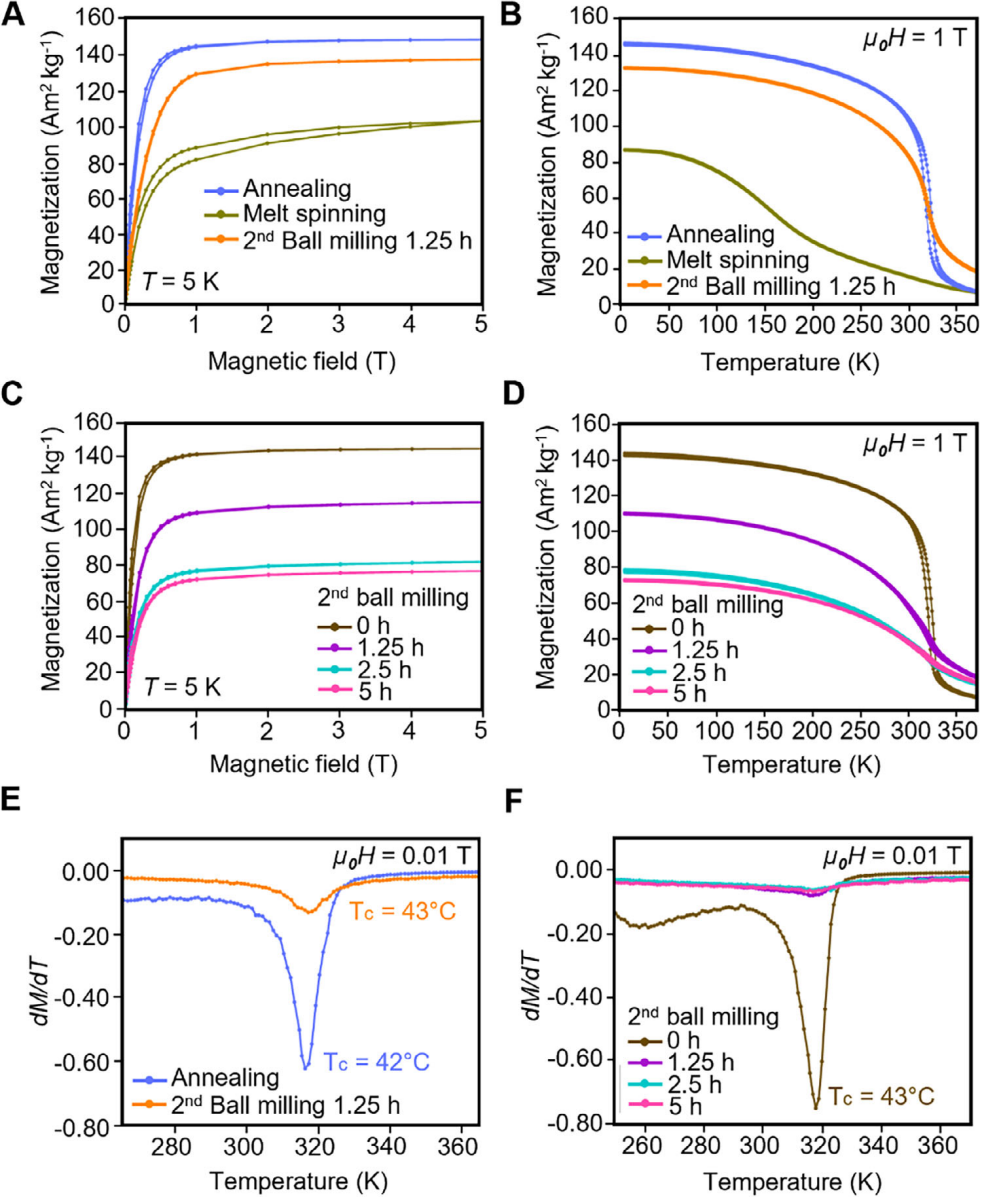
Magnetic properties of Mn_0.65_Fe_1.30_P_0.65_Si_0.37_ samples synthesized via different fabrication processes. The Mn_0.65_Fe_1.30_P_0.65_Si_0.37_ sample corresponds to the following synthesis stages: melt spinning (Stage II), heat treatment (annealing, Stage III), and a second surfactant‐assisted ball milling (Stage IV). A) Isothermal field‐dependent magnetization (*M‐H*) curves measured at 5 K and B) Temperature‐dependent magnetization (*M‐T*) curves measured at 1 Tesla (T) for samples subjected to different synthesis stages. C) *M‐H* curves at 5 K and D) *M‐T* curves at 1 T for re‐synthesized samples subjected to 2nd ball milling (Stage IV) for durations of 1.25, 2.5, and 5 h. E) Derivative of magnetization as a function of temperature (*dM/dT*) for the samples after annealing (Stage III) and a 2nd ball milling (Stage IV). F) *dM/dT* for re‐synthesized samples when subjected to varying durations of 2nd milling (Stage IV).

The magnetic properties of Mn_0.65_Fe_1.30_P_0.65_Si_0.37_ particles at various synthesis stages are presented in **Figure** **3**. The magnetic field dependence of magnetization (*M‐H*) curves at 5 K corresponding to different processing steps is shown in Figure 3A and Table S1 (Supporting Information). The melt‐spun sample exhibits a saturation magnetization of 103 Am^2^ kg^−1^. The initial rapid rise in magnetization, followed by a more gradual increase beyond 1 T, indicates incomplete magnetic saturation, likely due to the presence of impurity phases—as confirmed by XRD analysis of the melt‐spun material. Following annealing at 1100 °C, the saturation magnetization increases markedly to 149 Am^2^ kg^−1^. This enhancement is attributed to the elimination of impurity phases through annealing and grain growth in the magnetic particles. Larger grains promote improved magnetic ordering, which enhances saturation magnetization by reducing grain boundary effects and strengthening domain alignment.^[^ ^58^ ^]^ However, after the second ball‐milling process (1.25 h), the saturation magnetization slightly decreases to 138 Am^2^ kg^−1^. This reduction is primarily attributed to the introduction of microstrain and mechanical stress during milling, which induce crystal lattice distortions and disrupt magnetic domain alignment, thereby diminishing the overall magnetic performance. The temperature‐dependent magnetization (*M‐T*) curves of the Mn_0.65_Fe_1.30_P_0.65_Si_0.37_ sample, measured under an applied magnetic field of 1 T at various synthesis stages, are presented in Figure 3B. These curves reveal a first‐order magnetic phase transition from the ferromagnetic to the paramagnetic state near the Curie temperature. The melt‐spun sample shows a gradual decline in magnetization with increasing temperature, indicating a relatively broad transition region. Following the annealing process, the phase transition becomes significantly sharper, as evidenced by a distinct and abrupt drop in magnetization near the Curie temperature. This sharpening of the transition confirms that annealing and grain growth not only enhance the overall magnetization but also improve the definition of the magnetic phase transition. In contrast, a slight broadening of the phase transition is observed after the second ball milling (1.25 h).

The influence of further increasing second ball milling durations on the magnetization behavior of the re‐synthesized sample is shown in Figure 3C and Table S2 (Supporting Information). A consistent decrease in magnetization is observed with increasing milling time. The saturation magnetization at 5 K decreases from 146 Am^2^ kg^−1^ in the annealed state to 77 Am^2^ kg^−1^ after 5 h of milling. This decline is primarily attributed to the spin‐canting effect, which becomes increasingly significant as particle size decreases. In this phenomenon, spins at or near the nanoparticle surface become misaligned due to surface anisotropy and structural disorder, thereby reducing the net magnetization.^[^ ^59^ ^]^ Additionally, the progressive reduction in crystallite size due to prolonged milling can result in the gradual loss of long‐range ferromagnetic order. This structural degradation further contributes to the observed decrease in saturation magnetization.^[^ ^43^ ^]^ As shown in Figure 3D, increasing the duration of second ball milling from 1.25 to 5 h results in a gradual broadening of the magnetic phase transition. Prolonged milling diminishes the sharp transition characteristic of bulk materials, leading to a significantly broadened transition region. This broadening is primarily attributed to the accumulation of microstrain, along with the reduction in crystallite size during milling.^[^ ^60^ ^]^ The structural imperfections distort the crystal lattice and disrupt magnetic domain alignment, weakening magnetic ordering and thereby reducing the sharpness of the transition. A similar trend of reduced magnetization and a broadened first‐order magnetic phase transition with extended milling durations has also been reported in the literature.^[^ ^43^, ^61^ ^]^

The Curie temperature (*T_c_*) is determined by using the Knee method^[^ ^62^ ^]^ from the temperature‐dependent magnetization (*M‐T*) curves measured under a low magnetic field of 0.01 T (Figure 3E,F). The Curie temperature of the synthesized MCM particles is identified at the point where the temperature derivative of magnetization (*dM/dT*) reaches its minimum slope, indicating the onset of the magnetic phase transition. In the case of the melt‐spun sample, the *M‐T* curve lacks a distinct and sharp first‐order magnetic phase transition, making the precise determination of Curie temperature unreliable; hence, this sample is excluded from the Curie temperature analysis. As shown in Figure 3E, the obtained Curie temperatures for both the annealed and second ball‐milled samples fall within the range of 42 °C (316 K) to 43 °C (317 K), respectively. Furthermore, as illustrated in Figure 3F, the Curie temperature exhibits only a slight decrease with increasing ball‐milling duration (0–5 h), shifting marginally from 43 °C (317 K) to 42 °C (316 K). This slight reduction in Curie temperature is likely due to the introduction of microstrain and alterations in magnetic interactions caused by prolonged milling. Additionally, compared with Gd‐based magnetocaloric materials, which undergo significant T_c_ reduction upon mechanical milling,^[^ ^39^ ^]^ the (Mn,Fe)_2_(P,Si) system exhibits superior stability against size‐induced T_c_ reduction. This enhanced stability is a highly desirable feature for future integration into miniaturized thermomagnetic biomedical devices, where maintaining consistent magnetothermal performance upon downsizing is critical.

In addition, the magnetization behavior of Mn_0.65_Fe_1.30_P_0.65_Si_0.37_ particles is further investigated in comparison with iron (II, III) oxide (Fe_3_O_4_) and carbonyl iron powder (CIP), two commonly used magnetic materials in biomedical applications (i.e., implantable MEMS‐based devices).^[^ ^55^ ^]^ Fe_3_O_4_ is one of the most extensively studied and is approved by the U.S. FDA for use in biomedical applications such as MRI contrast agents, drug delivery systems, and thermal‐based therapies.^[^ ^63^, ^64^ ^]^ Although CIP is not FDA‐approved for biomedical applications beyond oral supplementation,^[^ ^65^ ^]^ it has garnered considerable research interest due to its high magnetization, positioning it as a promising candidate for magnetic hyperthermia^[^ ^65^ ^]^ and magnetically actuated devices.^[^ ^55^ ^]^ The magnetization behavior (*M‐H* curves) of three magnetic particles—CIP, Fe_3_O_4_, and Mn_0.65_Fe_1.30_P_0.65_Si_0.37_ (MCM)—is assessed at 310 K (37 °C) (Figure S4, Supporting Information). The saturation magnetization of MCM is measured to be 76.83 Am^2^ kg^−1^, which is lower than that of CIP (204.93 Am^2^ kg^−^1), but comparable to that of Fe_3_O_4_ (78.57 Am^2^ kg^−1^). As shown in Figure S4 (Supporting Information), within the low magnetic field range of −20–20 mT, Fe_3_O_4_ exhibits a noticeably larger hysteresis loop compared to both the MCM and CIP samples. This broader loop indicates greater coercivity in Fe_3_O_4_, suggesting higher energy barriers for magnetization reversal. Such characteristics may limit its responsiveness to moderate field strengths, potentially hindering efficient magnetic switching or heating performance in low‐field applications. Additionally, these findings of Fe_3_O_4_ align with previously reported values, where Fe_3_O_4_ used in hyperthermia applications demonstrates saturation magnetizations of ≈40 Am^2^ kg^−1^ at 300 K^[^ ^66^ ^]^ and 73 Am^2^ kg^−1^ at 300 K.^[^ ^67^ ^]^ This comparison suggests that the MCM sample maintains sufficient magnetization for heating applications under alternating magnetic field exposure, on par with widely used magnetic materials such as Fe_3_O_4_. It is worth noting that the saturation magnetization values and overall magnetic behaviors of Fe_3_O_4_ can largely be attributed to particle size and the associated magnetic state. Previous studies utilized Fe_3_O_4_ nanoparticles with average diameters in the range of 8–15 nm,^[^ ^66^, ^67^ ^]^ where the particles typically exhibit superparamagnetic behavior. In contrast, Fe_3_O_4_ particles in the micrometer range (1–5 µm), as used in this study, retain ferrimagnetic ordering, which results in different magnetic characteristics, including higher coercivity and remanence.

### 2.3 Fabrication and Thermal Characterization of Mn_0.65_Fe_1.30_P_0.65_Si_0.37_ Wax Composite

In magnetothermal applications, wax absorbs the heat generated by magnetic particles under an alternating magnetic field and stores it as latent heat during its solid‐to‐liquid phase transition. This thermally induced phase change can be strategically harnessed to trigger the transformation of wax from a solid to a liquid state, enabling the controlled release of encapsulated therapeutic agents at targeted sites within the body. In particular, lanolin wax and bayberry wax (BBW) (Figure S5, Supporting Information) are chosen for their favorable physicochemical properties, including biocompatibility and appropriate melting points. In this study, commercial lanolin wax with a measured melting point of 36 °C is used, along with BBW, which exhibits a melting point range of 39–49 °C.

The wax matrices are prepared in different volume fractions from 30 to 36 vol.% BBW to achieve an optimal melting point. This target is set at ≈42 °C to ensure the matrix remains within the physiologically safe temperature range, safely below the threshold for cellular damage, while ensuring the synthesized magnetocaloric particle generates sufficient heat to trigger phase change of the wax before reaching its Curie temperature. The thermal properties of the wax matrix are evaluated using differential scanning calorimetry (DSC). The second heating cycle of all synthesized wax matrices is recorded (Figure S6, Supporting Information). Melting points are determined and are presented in **Table** **3**. The results show that increasing the BBW fraction led to an increase in melting point, with the highest value observed for 36 vol.% BBW at 46.4 °C and the lowest value for 30 vol.% BBW at 42.5 °C. Owing to its optimal melting point, which closely aligns with the measured Curie temperature (43 °C), the 30 vol.% bayberry wax (BBW) matrix is selected as the binder for the magnetocaloric wax composite and further evaluated through inductive heating tests under exposure to an alternating magnetic field (AMF).

**Table 3:** Thermal parameters of the different wax matrix compositions by volume of bayberry wax obtained from the DSC analysis.

Bayberry wax (BBW) [vol.%]	Melting temperature, *T_melting_* [°C]
30	42.5
32	45.2
34	46.2
36	46.4

In addition to the heating behavior observed in the differential scanning calorimetry (DSC) measurements (Figure S6, Supporting Information), multiple endothermic peaks are detected across all samples. These peaks indicate the melting of distinct phases, a phenomenon likely attributed to the multi‐component composition of natural waxes, in which different chemical constituents, such as fatty acids and wax esters, undergo phase transitions at varying temperatures.^[^ ^68^ ^]^ This thermal complexity underscores the importance of considering a spectrum of thermal transition events rather than relying on a single melting point, particularly when designing thermally responsive systems for biomedical applications.

### 2.4 Magnetic Heating Performance of Magnetic Wax Composite under an Alternating Magnetic Field (AMF)

Developing magnetic materials with self‐limiting heating behavior presents a promising approach for enhancing the safety of biomedical heating systems. As previously discussed, this intrinsic “switch‐off” functionality can be achieved by tuning the material's Curie temperature (*T_c_*). The self‐regulation mechanism typically unfolds in three distinct stages: i) an initial rapid temperature rise driven by efficient magnetic energy dissipation when the particles are well below their *T_c_*; ii) a slower rate of temperature increase as the material approaches *T_c_*, reflecting a decline in both magnetization and magnetic susceptibility; and iii) thermal stabilization near or slightly above Curie temperature, resulting in a substantial reduction in heat generation under the applied alternating magnetic field. To evaluate the self‐limiting heating behavior and heating efficiency of the tailored Mn_0.65_Fe_1.30_P_0.65_Si_0.37_ (MCM) particles, comparative inductive heating experiments are conducted alongside carbonyl iron powder (CIP) and iron (II, III) oxide (Fe_3_O_4_). Each material is dispersed in water at a concentration of 15 vol.% and exposed to an alternating magnetic field of 10 mT at 244 kHz at room temperature for a duration of 15 min.

**Figure 4:**
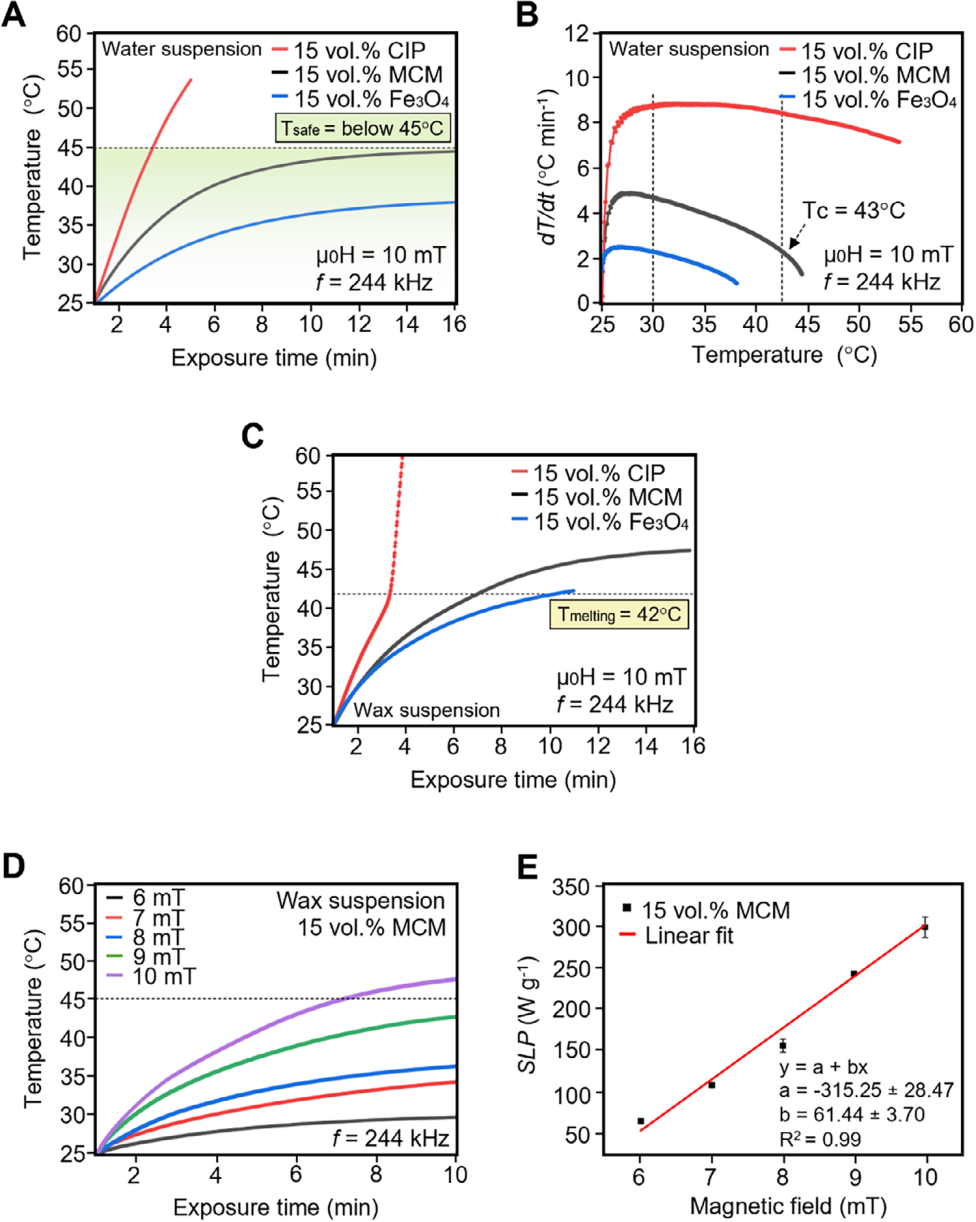
A) Heating profiles and B) corresponding heating rates (dT/dt) for three magnetic samples, iron (II, III) oxide (Fe_3_O_4_), carbonyl iron powder (CIP), and Mn_0.65_Fe_1.30_P_0.65_Si_0.37_ (MCM), each dispersed in water at a particle concentration of 15 vol.% and measured under an alternating magnetic field (AMF) of 10 mT at 244 kHz. C) Heating profiles for the same magnetic particles encapsulated in a wax matrix at 15 vol.% concentration under identical AMF conditions measured under 10 mT at 244 kHz. D) Heating profile and E) specific loss power (*SLP*) values of MCM samples with 15 vol.% particle concentration when subjected to varying field amplitudes (6–10 mT) at a constant frequency of 244 kHz.

As shown in **Figure** **4**A, the 15 vol.% Fe_3_O_4_ suspension exhibits temperature saturation below 45 °C, where the temperature is plateauing ≈37 °C. However, this stabilization is not the result of an intrinsic self‐regulating mechanism related to its Curie temperature, as Fe_3_O_4_ possesses a high *T_c_* of ≈585 °C.^[^ ^69^ ^]^ Rather, the limited thermal response arises from the material's low magnetic responsiveness, resulting in inefficient dissipation of magnetic energy into heat. It is important to note that heat dissipation by magnetic particles in thermal applications, such as magnetic hyperthermia, is strongly influenced by dipolar interactions and the amplitude of the applied alternating magnetic field (AMF). At higher particle concentrations, reduced interparticle distances enhance dipolar coupling,^[^ ^70^ ^]^ promoting ferromagnetic interactions between adjacent particles.^[^ ^71^ ^]^ This collective magnetic behavior increases energy dissipation. Therefore, the heating performance of Fe_3_O_4_ can be improved by adjusting the field strength or particle concentration. In contrast, under the same AMF conditions and particle concentration, the CIP suspension heats rapidly, reaching 40 °C within 2 min 42 s (Figure 4A). This response is consistent with the high magnetic saturation, high permeability, and lower coercivity of pure iron,^[^ ^72^ ^]^ which facilitates more efficient energy dissipation compared to Fe_3_O_4_. However, CIP lacks an intrinsic thermal regulation mechanism, resulting in continuous temperature rise beyond the physiologically safe threshold, exceeding 55 °C within just 3 min 27 s. These observations highlight a fundamental trade‐off between heating efficiency and thermal safety. While materials such as CIP provide high heating rates, they pose a risk of overheating without self‐limiting features. This underscores the critical importance of precise Curie temperature tuning in magnetothermal agents to achieve both effective heat generation and safe, self‐regulated thermal behavior in biomedical applications.

The 15 vol.% MCM suspension demonstrates a good balance between heating performance and thermal safety. As illustrated in Figure 4A, the suspension exhibits a rapid temperature rise to 40 °C within 6 min, indicating superior heating efficiency relative to the Fe_3_O_4_ suspension at the same volumetric concentration, which fails to achieve comparable temperatures. This enhanced thermal response is attributed to increased magnetic losses below the Curie temperature and the material's heightened sensitivity to the applied field, a result of its intrinsic magnetocaloric properties. Although the heating efficiency of MCM is lower than that of CIP, it uniquely offers self‐regulating thermal behavior. Upon exceeding its *T_c_* (43 °C), the system stabilizes its temperature, thereby maintaining thermal levels within the physiologically safe range (below 45 °C). This self‐limiting characteristic is governed by the sharp change in magnetization near *T_c_*, induced by the first‐order magnetic phase transition inherent to giant magnetocaloric effect materials. This transition is further corroborated by the temperature‐dependent magnetization profile (Figure S7, Supporting Information), which reveals a pronounced drop in magnetization near its Curie temperature, confirming the material's transition from a ferromagnetic to a paramagnetic state. It is worth noting that a slight temperature overshoot above *T_c_* is observed, likely due to a transient lag in the phase transition kinetics across the particle ensemble, which may momentarily delay the onset of thermal stabilization.

The heating rate (*dT/dt*) of the MCM suspension provides further insight into its thermal behavior, particularly in relation to its intrinsic self‐regulating thermal capability. As presented in Figure 4B, the MCM suspension exhibits an initial heating rate of ≈5 °C min^−1^, indicating a notably higher heating efficiency than the Fe_3_O_4_ suspension, which displays a maximum rate of only 2.5 °C min^−1^. This disparity reflects the limited heat generation capacity of Fe_3_O_4_, primarily due to its lower magnetic responsiveness under the same applied magnetic field conditions. Importantly, the heating rate of the MCM suspension progressively decreases as the temperature approaches its Curie temperature, clearly illustrating its efficient and precise self‐regulating thermal behavior. While the initial heating rate of the MCM suspension is lower than that of the CIP suspension—which reaches a peak rate of ≈9 °C min^−1^, the CIP suspension lacks the intrinsic thermal regulation exhibited by MCM. This uncontrolled thermal behavior underscores the absence of self‐limiting mechanisms in CIP and raises potential safety concerns for biomedical applications that require precise thermal control.

The thermal response of the magnetic wax composite is further examined to evaluate the capability of each magnetic particle to induce the phase transition of the wax matrix within physiologically safe temperature limits. Composites containing 15 vol.% magnetic particles are exposed to an alternating magnetic field at 10 mT and 244 kHz, with temperature evolution monitored over time (Figure 4C). Among the tested samples, the Fe_3_O_4_ composite reaches the wax melting point of 42 °C in 9 min 17 s, reflecting slow heat generation, consistent with its limited performance observed in aqueous suspension. In contrast, the CIP composite reaches 42 °C within just 2 min 24 s. However, it exhibits uncontrolled heating, rapidly surpassing 45 °C in ≈3 min, thereby confirming the absence of intrinsic thermal regulation. In comparison, the 15 vol.% MCM wax composite reaches 42 °C in 6 min and 14 s, followed by thermal stabilization ≈47 °C. It is worth noting that the wax composite exhibits a higher temperature threshold compared to the aqueous suspension in Figure 4A. As a phase change material (PCM), wax absorbs latent heat during its melting process, maintaining a nearly constant temperature throughout the phase transition. Once the wax is fully melted, further energy input causes the temperature to rise again. In the case of the CIP–wax composite (Figure 4C), a rapid and sudden temperature rise is observed after surpassing the melting point of ≈42 °C, in contrast to the more gradual heating profile of the CIP sample dispersed in water (Figure 4A). The higher temperature threshold observed in the wax‐based samples is likely attributed to the lower heat capacity of wax compared to water. This lower heat capacity enables a faster temperature increase under continued magnetic heating, potentially resulting in higher final temperatures than those observed in aqueous systems. Notably, the MCM sample dispersed in water (Figure 4A) presents a thermal response that is likely relevant to in vivo biological conditions, given that water is the primary constituent of biological fluids. In such aqueous systems, the temperature rise is inherently self‐limiting near 43 °C, remaining within the physiologically safe range and below the critical threshold of 45 °C associated with tissue damage. While the thermal overshoot observed in wax‐based composites reflects distinct heating behavior, it is unlikely to pose a risk in in vivo applications. Although the current thermal characterization is conducted in water, which provides a reasonable approximation of biological environments, further validation in physiologically relevant media such as phosphate‐buffered saline (PBS) would enhance the translational relevance of these findings.

Specific loss power (*SLP*) quantifies the thermal energy generated per unit mass of magnetic material under exposure to an alternating magnetic field, with higher *SLP* values indicating superior heating efficiency. Since *SLP* is influenced by both the amplitude and frequency of the applied field,^[^ ^66^ ^]^ the field‐dependent heating behavior of the 15 vol.% MCM composite is evaluated under alternating magnetic field strengths ranging from 6 to 10 mT at a fixed frequency of 244 kHz (Figure 4D). *SLP* values are calculated using Equation (3) in the Experimental Section, with the corresponding parameters summarized in Table S3 (Supporting Information). As shown in Figure 4D,E, both temperatures increase and *SLP* values rise linearly with increasing field amplitude. Specifically, the *SLP* values increase from 65 W g^−1^ at 6 mT to 297 W g^−1^ at 10 mT. The observed enhancement is attributed to intensified magnetic dipole–dipole interactions and more effective magnetic moment alignment at higher field strengths, which collectively improve energy conversion efficiency.^[^ ^73^ ^]^ As discussed earlier, the irregularities in shape observed in MCM particles can influence their magnetothermal performance. While measurements averaged over many particles appear consistent and reproducible, individual particles may heat differently, leading to uneven temperature distributions at the microscopic scale. Nevertheless, the MCM composite with a Curie temperature of 317 K lies well within the physiologically acceptable range for biomedical hyperthermia and demonstrates a self‐regulated heating response under alternating magnetic fields (AMFs). Further optimization of AMF parameters and morphological control across both bulk and nanoscale regimes will be essential for enhancing magnetothermal precision and tunability.

In addition to its favorable self‐regulated temperature control and promising heating performance, the magnetocaloric material studied here shows potential for application in magnetothermal particle thermometry (MPT), a technique for direct temperature measurement based on the temperature‐dependent magnetic response of the particles. In MPT, thermal sensing is achieved by analyzing the voltage induced in a pickup coil as magnetic particles undergo cyclic magnetization under exposure to an alternating magnetic field. Magnetic materials with low Curie temperatures have been reported to enhance thermometric sensitivity.^[^ ^69^ ^]^ Due to the sharp change in total magnetic induction *B*(*t*) =  μ_0_[*H*(*t*) + *M*(*t*)], near *T_c_*. Below *T_c_*, the magnetization *M*(*t*) is high, while above *T_c_*, it decreases sharply as the material transitions to a paramagnetic state. This abrupt change results in a distinguishable variation in the induced voltage signal, allowing accurate extraction of local temperature information. Thus, the Mn_0.65_Fe_1.30_P_0.65_Si_0.37_ composite may also serve as a multifunctional platform for both controlled thermal actuation and real‐time temperature sensing in biomedical environments.

### 2.5 In Vitro Evaluation of Biocompatibility and Biodegradability of Magnetocaloric Wax‐based Composite

**Figure 5:**
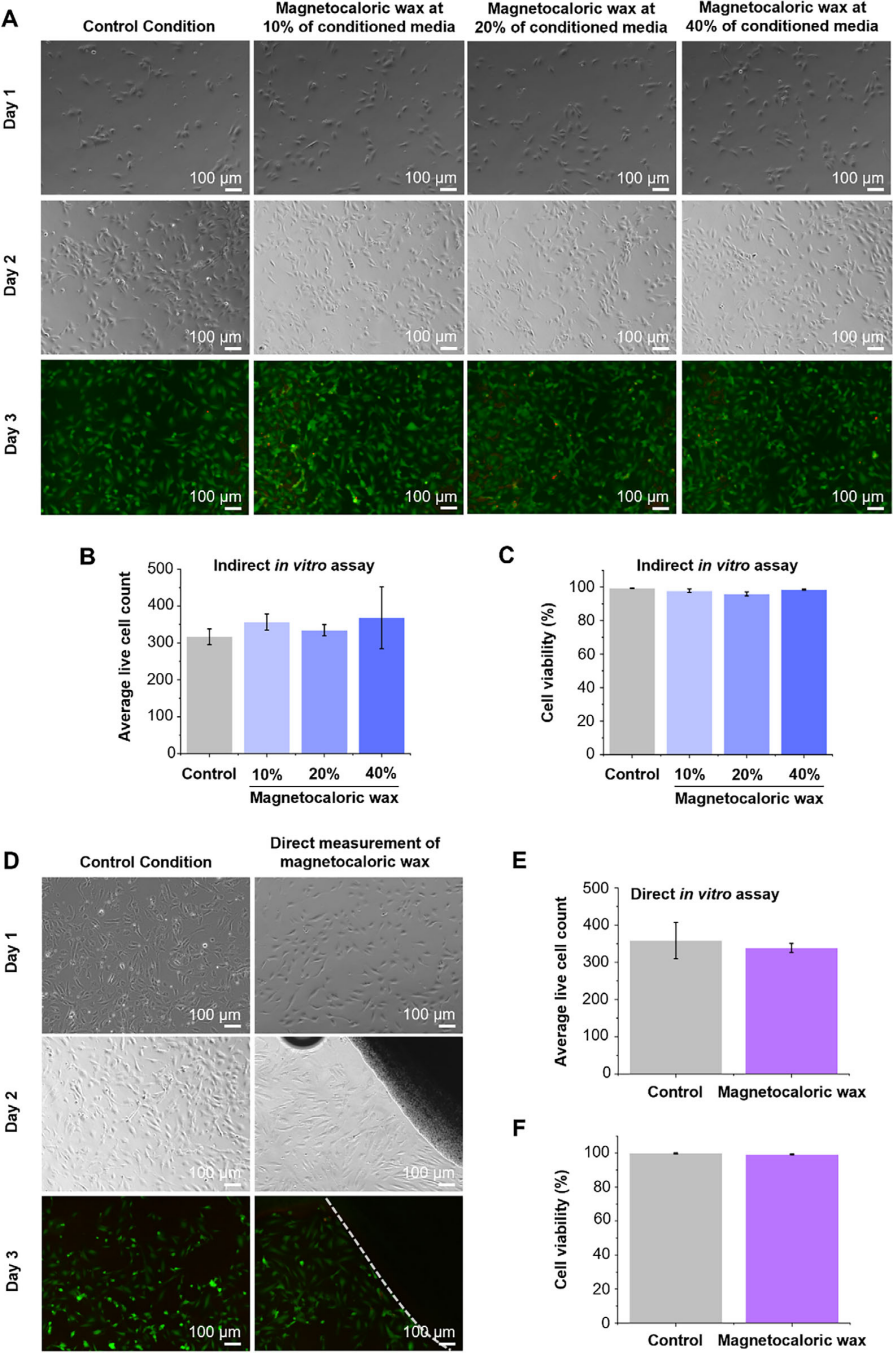
In vitro assessment of the biocompatibility of MCM wax composites containing 15 vol.% Mn_0.65_Fe_1.30_P_0.65_Si_0.37_ (MCM) particles. A) Representative images showing cell viability of HUVECs treated with 10%, 20%, and 40% conditioned media (Indirect in vitro assay). Images captured on day 3 demonstrate cell morphology and viability under different treatment conditions (*n* = 2). Scale bar = 100 µm. B) Average live cell count and C) Percentage of cell viability of HUVECs across varying conditioned media (10%, 20%, and 40%) on day 3 (*n* = 2). D) Representative images of HUVECs directly exposed to the magnetocaloric wax composites (Direct in vitro assay). Live/Dead staining images on day 3 illustrate the impact on cell morphology and viability (*n* = 2). Scale bar = 100 µm. The outer boundary of the wax composite is indicated by the white dashed line. E) Average live cell count and F) Percentage of cell viability of HUVECs in direct contact with the wax composite on day 3 (*n* = 2).

The biocompatibility of the magnetocaloric wax composite is a key consideration for its application in implantable and bioresorbable medical devices. To assess potential cytotoxic effects, indirect in vitro assays are initially performed using human umbilical vein endothelial cells (HUVECs) exposed to conditioned media obtained from the wax composite with 15 vol.% Mn_0.65_Fe_1.30_P_0.65_Si_0.37_ particles. The wax composites are incubated in endothelial growth medium (EGM) at 37 °C for 7 days. Following incubation, the resulting media is diluted with normal EGM to prepare 10%, 20%, and 40% conditioned media solutions. These formulations are then applied to HUVECs and maintained for a total of 3 days. To further investigate the underlying mechanism of any observed cytotoxicity, Live/Dead cell staining is performed on day 3. As shown in **Figure** **5**A, live cells exhibit green fluorescence (calcein‐AM), whereas dead cells exhibit red fluorescence (ethidium homodimer‐1). Over a three‐day incubation period, cells exposed to 10%, 20%, and 40% conditioned media exhibit fluorescence patterns similar to those of the untreated control group. Specifically, bright green fluorescence is predominantly observed over minimal red fluorescence, suggesting predominantly viable cells. Quantitative analyses of live cell counts and viability percentages (Figure 5B,C) support these findings, demonstrating comparable cell growth and cell viability (>90%) across all treatment groups and controls. To complement these results, direct contact in vitro biocompatibility assays are also conducted, where the magnetocaloric composite is placed in close proximity to HUVECs. As shown in Figure 5D, Live/Dead staining again reveals a predominance of green fluorescence on day 3, indicating preserved cell viability. Quantitative analyses (Figure 5E,F) show that both live cell counts and viability percentages (>90%) upon exposure to the wax composite remain comparable to those of the control group. Collectively, these results confirm that the magnetocaloric wax composite is biocompatible and non‐cytotoxic under both indirect and direct exposure conditions.

The observed non‐cytotoxic nature of the magnetocaloric Mn_0.65_Fe_1.30_P_0.65_Si_0.37_ particles is attributed to its biologically constituent elements—manganese (Mn), iron (Fe), phosphorus (P), and silicon (Si)—all of which are naturally present in the human body and are essential for key physiological functions. Manganese plays a critical role in enzymatic processes, bone formation, and blood coagulation,^[^ ^74^ ^]^ with an FDA‐recommended daily intake (RDI) of 2–5 mg for adults.^[^ ^75^ ^]^ It is primarily absorbed in the form of Mn^2+^ and Mn^3+^.^[^ ^76^ ^]^ Excess manganese is excreted via bile.^[^ ^77^ ^]^ Iron, which exists primarily in the form of Fe^3+^ and Fe^2+^, is stored in proteins such as hemoglobin, myoglobin, transferrin, and ferritin, and is essential for homeostasis.^[^ ^78^ ^]^ The average adult body contains ≈4 g of Fe,^[^ ^79^ ^]^ with a recommended dietary allowance (RDA) of 8 mg day^−1^ for males and 18 mg day^−1^ for females. However, only 1–2 mg is typically absorbed via the intestinal tract.^[^ ^78^ ^]^ Excess iron is stored in cytosolic ferritin complexes,^[^ ^80^ ^]^ and dietary Fe^3+^ is later reduced to Fe^2+^ in the intestine for absorption.^[^ ^81^ ^]^ Phosphorus plays a central role in DNA and RNA synthesis, cellular metabolism, and ATP‐mediated energy transfer.^[^ ^82^, ^83^ ^]^ Once absorbed, phosphorus exists predominantly as phosphate ions (PO_4_^3−^), which combine with calcium to form hydroxyapatite (Ca_10_(PO_4_)_6_(OH)_2_), the main inorganic constituent of bone.^[^ ^84^, ^85^, ^86^ ^]^ Around 90% of total body phosphate is stored in the skeleton, with the remainder distributed across soft tissues and extracellular fluids.^[^ ^87^ ^]^ The RDA for phosphorus in adults is 700 mg day^−1^,^[^ ^88^ ^]^ and it is primarily absorbed in the jejunum before being excreted via the feces.^[^ ^85^ ^]^ Silicon, contributes to bone mineralization and collagen synthesis, playing a key role in maintaining connective tissue integrity.^[^ ^89^ ^]^ The typical dietary intake ranges from 19 to 31 mg day^−1^, mostly in the form of orthosilicic acid (Si(OH)_4_),^[^ ^90^ ^]^ which is filtered by the kidneys and excreted in urine.^[^ ^91^, ^92^, ^93^ ^]^ Taken together, the elemental composition of Mn_0.65_Fe_1.30_P_0.65_Si_0.37_ particles is well‐aligned with the requirements for in vivo biocompatibility. The incorporation of naturally metabolizable and physiologically regulated elements strongly supports the non‐toxic nature of the composite and highlights its potential for future biomedical applications involving localized thermal actuation.

The biodegradability assessment of the magnetocaloric wax composite in phosphate‐buffered saline (PBS) at 37 °C over a 4‐week period (Figure S8, Supporting Information) demonstrates that the wax matrix retained its structural integrity, exhibiting negligible weight loss, over 90% mass retained, as calculated using Equation (4) in the Experimental section. This is attributed to its inherent hydrophobicity. This resistance to degradation in aqueous environments confirms the composite's low water permeability and stability under physiological conditions. Furthermore, visual and physical analysis (Figure S8, Supporting Information) confirm that the magnetocaloric particles remained effectively encapsulated within the wax matrix throughout the study period. Although the composite does not undergo significant biodegradation, its ability to preserve particle encapsulation over time suggests its potential suitability as a carrier for drug delivery applications, where sustained protection and controlled release of therapeutic agents are required.

## 3 Conclusion

In summary, we have demonstrated the first implementation of a (Mn,Fe)_2_(P,Si)‐based magnetocaloric compound, Mn_0.65_Fe_1.30_P_0.65_Si_0.37_ (MCM), for self‐regulating magnetothermal applications in biomedical settings. By exploiting its tunable Curie temperature (T_c_) to achieve intrinsic temperature self‐limitation near the physiological safety threshold, the tailored magnetic behavior offers a promising strategy for minimizing the risk of overheating during thermal treatments, thereby enhancing the safety profile of magnetic particle‐based thermal therapies. Synthesized via high‐energy ball milling, melt spinning, and heat treatment, the compound exhibits a sharp and tunable Curie temperature (*T_c_*) at 43 °C—strategically aligned with the physiological safety threshold (below 45 °C). This will minimize the risk of overheating surrounding healthy tissues during thermal stimulation. Micron‐sized MCM particles (≈3 µm) are incorporated into both aqueous suspensions and a wax matrix to assess heating performance under alternating magnetic fields (AMFs). When subjected to a 10 mT AMF at 244 kHz, a 15 vol.% aqueous suspension exhibits self‐limiting heating, with the temperature stabilizing just below 45 °C. This response confirms the compound's intrinsic thermal “switch‐off” mechanism governed by its precisely tuned Curie temperature. Similarly, the MCM‐wax composite reaches the wax melting point in 6 min and 14 s, initiating a solid‐to‐liquid phase transition, followed by thermal stabilization near 47 °C. The slightly higher stabilization temperature observed in the wax system is attributed to its inherently lower heat capacity in comparison with water. Field‐dependent heating studies conducted under alternating magnetic field amplitudes ranging from 6 to 10 mT demonstrate a linear increase in both temperature and specific loss power (*SLP*), highlighting the compound's efficient energy conversion. In vitro biocompatibility, asessed via Live/Dead cell staining assays of human umbilical vein endothelial cells (HUVECs), shows no significant cytotoxicity, thereby confirming the material's therapeutic safety. Biodegradability assessments of the wax composite further confirm its hydrophobic nature and ability to retain encapsulated magnetocaloric particles over 4 weeks in PBS at 37 °C, demonstrating structural stability and potential for long‐term delivery applications. Future work will investigate AMF‐induced cytotoxicity to assess dynamic responses under magnetothermal stimulation.

Collectively, these findings establish Mn_0.65_Fe_1.30_P_0.65_Si_0.37_ as a multifunctional material that provides precise and non‐invasive thermal regulation through Curie temperature, exhibits comparable magnetization, ensures efficient heat generation under moderate alternating magnetic field exposure, and demonstrates favorable biocompatibility and biodegradability. This comprehensive profile highlights its potential as a strong candidate for next‐generation biomedical technologies—including hyperthermia therapy, thermally triggered drug delivery systems, magnetothermal MEMS‐based soft actuators, and self‐regulated thermally induced stimulation systems—where both therapeutic efficacy and patient safety are crucial.

## 4 Experimental Section

### Synthesis of Bulk (Mn, Fe)_2_(P, Si)‐Based Compound

The bulk magnetocaloric (Mn, Fe)_2_(P, Si)‐based compound was synthesized through a multi‐step process involving ball milling, melt spinning, and heat treatment (Figure S1, Supporting Information). Mn (99.7%), Fe (99.8%), Si (99.6%), and Fe_2_P powders (10 g total) were mixed and milled at 380 rpm for 10 h. The resulting fine powders were compacted into 10‐mm diameter pellets using a hydraulic press (1 GPa). These pellets were subsequently processed by melt spinning, wherein they were inserted into a quartz tube and melted using induction coils. The resulting molten metal was then pushed by gas pressure (P) through a small nozzle in the crucible onto a rotating copper wheel, where the liquid metal was rapidly cooled to form a solid ribbon. The ribbons were then sealed in ampoules under an Ar atmosphere and annealed at 1100 °C for 2 h. The annealed samples were rapidly quenched in water at room temperature. Subsequently, annealed samples (≈5 g) were milled again in the presence of 30 wt.% of oleic acid (as a surfactant) and 60 wt.% of heptane (solvent) for varying durations of 1.25, 2.5, and 5 h at 380 rpm. The mixture was dispersed in heptane solvent via ultrasonic vibration, followed by centrifugation to remove excess surfactant. Finally, the samples were dried in a vacuum oven at 70 °C for 24 h.

### Characterization Methods of (Mn, Fe)_2_(P, Si)‐Based Compound

X‐ray diffraction (XRD) patterns were collected at 80 °C using an Anton Paar TTK450 temperature chamber and a PANalytical X'Pert Pro diffractometer with Cu – K_α_ radiation. The XRD patterns were recorded within a 2θ range spanning from 20° to 90°. The XRD patterns were analyzed using Fullprof's implementation of the Rietveld refinement method. The average crystallite size of (Mn, Fe)_2_(P, Si)‐based samples was estimated based on the Scherrer equation (Equation 1), where *D_hkl_* is the average crystallite size, *hkl* are Miller indices of the crystal planes, *K* is the crystallite‐shape factor (0.9), λ is the wavelength of the X‐ray (0.154 nm for Cu – K_α_ radiation), *B_hkl_* is the full‐width at half‐maximum (FWHM) of the diffraction peak in radians, and θ is the Bragg angle. The full width at half‐maximum (*B_hkl_*) is determined before being applied in the Scherrer equation. To account for instrumental broadening, the *B_hkl_* value is obtained by subtracting the instrumental FWHM from the measured FWHM of the synthesized magnetic compound. The instrumental FWHM is calculated using Equation (2), based on the instrument‐specific parameters *U* = 0.09926, *V* = −0.1669, and *W* = 0.1004, obtained through calibration. The measured FWHM of the compound was extracted through Rietveld refinement of the obtained XRD data. In this study, the strongest XRD peak, which was the (111) reflection, was selected to estimate the crystallite size. Milled particle batches were examined by scanning electron microscopy (SEM) conducted on a JSM‐IT100. Field‐dependent magnetization (*M‐H*) and temperature‐dependent magnetization (*M‐T*) curves were measured using a superconducting quantum interference device (SQUID, Quantum Design MPMS 5XL) magnetometer.

(1) $$D_{hkl}=\;\frac{K\lambda }{B_{hkl}\cos\left(\theta \right)}$$

(2) $$FWHM^{2}=Utan^{2}\theta +Vtan\theta +W$$

### Preparation of Wax Matrix and Magnetic Wax Composite

A wax matrix (1 mL) was prepared by mixing bayberry wax (*Natural Heroes*) (0.3 mL) and lanolin wax (*Prounol*) (0.7 mL). The waxes were weighed in a glass vial, heated at 70 °C until fully liquefied, and then mixed using a vortex mixer at 2000 rpm for 120 s to achieve a homogeneous dispersion. The magnetic wax composites were fabricated by blending the wax matrix with magnetic particles, specifically Mn_0.65_Fe_1.30_P_0.65_Si_0.37_ magnetocaloric materials (MCM), carbonyl iron powder (CIP), and iron (II, III) oxide (Fe_3_O_4_). CIP used in this project is purchased from Sigma–Aldrich with cat.no. 44890 and its particle size was in the range of 1–10 µm. Similar to iron (II, III) oxide (Fe_3_O_4_), it was also purchased from Sigma–Aldrich with cat.no. 310069 and its particle size was ≈5 µm. Each composite (1 mL) contained 15 vol.% magnetic particles, corresponding to Mn_0.65_Fe_1.30_P_0.65_Si_0.37_ (1.049 g), CIP (1.179 g), and Fe_3_O_4_ (0.765 g). The liquefied wax matrix was combined with each type of magnetic particle and mixed using a vortex mixer at 2000 rpm for 120 s to ensure uniform dispersion.

### Characterization of Wax Matrix

Differential scanning calorimetry (DSC) was performed using a TA‐Q2000 DSC instrument equipped with a liquid nitrogen cooling system. Each sample was weighed (≈16–18 mg) and subjected to a series of heating and cooling cycles from −20 to 60 °C at a sweep rate of 20 K min^−1^. The samples were encapsulated in a Tzero aluminum pan with a punctured lid to facilitate water evaporation and the release of gas during thermal cycling.

### Inductive Heating of Magnetic Wax Composite under AMF

The heat generation efficiency of the magnetic particles dispersed in either water or the wax composite was evaluated under an alternating magnetic field (AMF) source (Magnetherm, nanoTherics Ltd.). The sample was tested under different AC magnetic field values (6–10 mT) at a constant frequency (*f* = 244 kHz). Each sample (1 mL) was placed at the center of the magnetic coil using a sample holder. Two glass optic fiber thermometers were inserted into the sample Eppendorf tube at the core and bottom of the sample. Prior to each measurement, a temperature equilibration was deemed achieved when the difference between the two probes remains within 0.5 °C for at least one minute before the start of recording. AMF heating procedures were recorded, with temperature measurements beginning 60 s before AMF activation and continuing until the set duration (10–15 min) was reached.

### Magnetic Particle Hyperthermia (MPH) Evaluation

The heating efficiency of the magnetocaloric particles was assessed using specific loss power (*SLP*), which quantifies the power absorption per unit mass of the magnetic material (W kg^−1^). The value is calculated by using Equation (3), where *C_s_* represents the specific heat capacity of the medium (assumed to be equal to that of water, *C_water_* = 4185 J L^−1^ °C^−1^), *m_Ps_* refers to the mass of magnetic particles within the 1 mL composite (g L^−1^), and *dT/dt* (°C s^−1^) corresponds to the initial rate of rapid temperature increase measured over the first 180 s following the activation of the alternating magnetic field.

(3) $$SLP=C_{water}\frac{dT}{dt}\frac{1}{m_{Ps}}$$

### Cell Culture

Indirect and direct measurements were employed to assess the potential cytotoxicity of leachable components from Mn_0.65_Fe_1.30_P_0.65_Si_0.37_ wax‐based composites containing 15 vol.% magnetocaloric particles. For the indirect test, ≈1 mL of the composite was incubated in EGM (211‐500, Merck KGaA, Darmstadt, Germany) supplemented with 10% fetal bovine serum (FBS) and 1% penicillin/streptomycin (P/S) at 37 °C for 7 days to allow the release of any soluble components. For the control condition, normal EGM was incubated at 37 °C for 7 days. Following incubation, on day 7, the extract solutions, containing potentially released compounds, were then collected and mixed with normal EGM at 10%, 20% and 40% to form the respective conditioned media. HUVECS were harvested using accutase (A6964, Sigma–Aldrich, Merck KGaA, Darmstadt, Germany) and resuspended in the respective conditioned media for replating in 24‐well plates (60 000 cells well^−1^). For the indirect test, cells were harvested using accutase and seeded in 24‐well plates (60 000 cells well^−1^), containing already the wax composites in the wells, using normal EGM. For both the indirect and direct tests, cells used in the experiments were in passage 5 (P5). Cells were cultured for 3 days in total and brightfield images were acquired every day using the Keyence BZ‐X800 microscope (Keyence Corporation, Osaka, Japan).

### In Vitro Cell Viability Assessment

After 3 days of culture, cell viability was assessed using a Live/Dead staining (LIVE/DEAD Cell Imaging Kit (488/570), R37601, Thermo Fisher Scientific, Rosny‐sous‐Bois, France) following the manufacturer's protocol. Briefly, cells were exposed to media containing the dye (Calcein AM and ethidium monodimer‐1) for 15 min in the dark at RT. Cells were then washed with PBS and imaged using the Keyence BZ‐X800 microscope. Results were reported as both the average number of live cells on day 3 (average number of cells calculated from images of 5 different areas of the well) and the percentage viability relative to untreated control cultures. The percentage viability was calculated by counting the number of live and dead cells and expressing the amount of live cells as a percentage of the total number of cells. Images were analyzed using ImageJ 1.54d (National Institute of Health, Bethesda, Maryland, USA).

### In Vitro Degradation Test

The degradability of the 30 vol.% wax matrix and Mn_0.65_Fe_1.30_P_0.65_Si_0.37_ wax composites containing 15 vol.% magnetocaloric particles was evaluated. Square samples (1 cm × 1 cm × 0.25 cm) were prepared by injecting the molten composite into custom‐designed 3D‐printed molds. The initial weights were 0.243 ± 0.001 g for the wax matrix samples and 0.263 ± 0.005 g for magnetocaloric wax samples. All samples were immersed in phosphate‐buffered saline (PBS) water (pH 7.4) at 37 °C. At designated time intervals (every 7 d), each sample was removed, rinsed with compressed air to eliminate surface moisture, and subsequently dried under nitrogen vacuum for 5 h to ensure complete removal of absorbed water. The sample was then left to dry overnight for an additional 2 days. The dry mass was recorded at each interval to track degradation progression. The percentage of weight loss was determined based on Equation (4), where *W_i_* denotes the initial dry weight of the sample before immersing it in the PBS condition and *W_f_* refers to the dry mass of the sample at each time point. The gradual loss of mass over time was used to quantify material degradation under simulated physiological conditions. Morphological changes, such as the formation of microcracks, and rheological alterations in the wax matrix were characterized at various stages of dissolution using optical microscopy.

(4) $$Weight\;loss\;\left(\%\right)=100-\;\frac{W_{i}-W_{f}}{W_{i}}\;x\;100$$

## Conflict of Interest

The authors declare no conflict of interest.

## Supporting information



Supporting Information

## Data Availability

The data that support the findings of this study are available from the corresponding author upon reasonable request.
