# Supplementary material for: Burn‐Safe Biodegradable Magnetocaloric Composites for Temperature‐Controlled Biomedical Applications
Source: Adv Sci (Weinh). 2025 Nov 25;13(15):e09914. doi: 10.1002/advs.202509914 (PMC13042379; doi:10.1002/advs.202509914)
Supplement: Supplementary file 1 — Supporting Information [file ADVS-13-e09914-s001.docx]

**Supporting Information**

**Burn-Safe Biodegradable Magnetocaloric Composites for Temperature-Controlled Biomedical Applications**

Pornpawee Uliss, Wuliji Hanggai, Friso Kahler, Elena Aprea, Qi Jia, Vasiliki Gkouzioti, Jean-Philippe Frimat, Ekkes Brück, and Clementine M. Boutry*

P. Uliss, F. Kahler, E. Aprea, C. M. Boutry

Faculty of Electrical Engineering, Mathematics and Computer Science (EEMCS), Department of Microelectronics (ME), Electronic Components, Technology and Materials (ECTM)

Delft University of Technology, 2628 CD Delft, the Netherlands

E-mail: [C.M.F.Viellard-Boutry@tudelft.nl](mailto:C.M.F.Viellard-Boutry@tudelft.nl)

W. Hanggai, E. Brück

Faculty of Applied Sciences, Department of Radiation Science and Technology (RST), Fundamental Aspects of Materials and Energy (FAME)

Delft University of Technology, 2628 CD Delft, the Netherlands

Q. Jia

Faculty of Applied Sciences, Department of Radiation Science and Technology (RST), Applied Radiation and Isotopes (ARI)

Delft University of Technology, 2628 CD Delft, the Netherlands

V. Gkouzioti, J.P. Frimat

Department of Neurology and Department of Human Genetics.

Leiden University Medical Center, 2333 ZC Leiden, the Netherlands


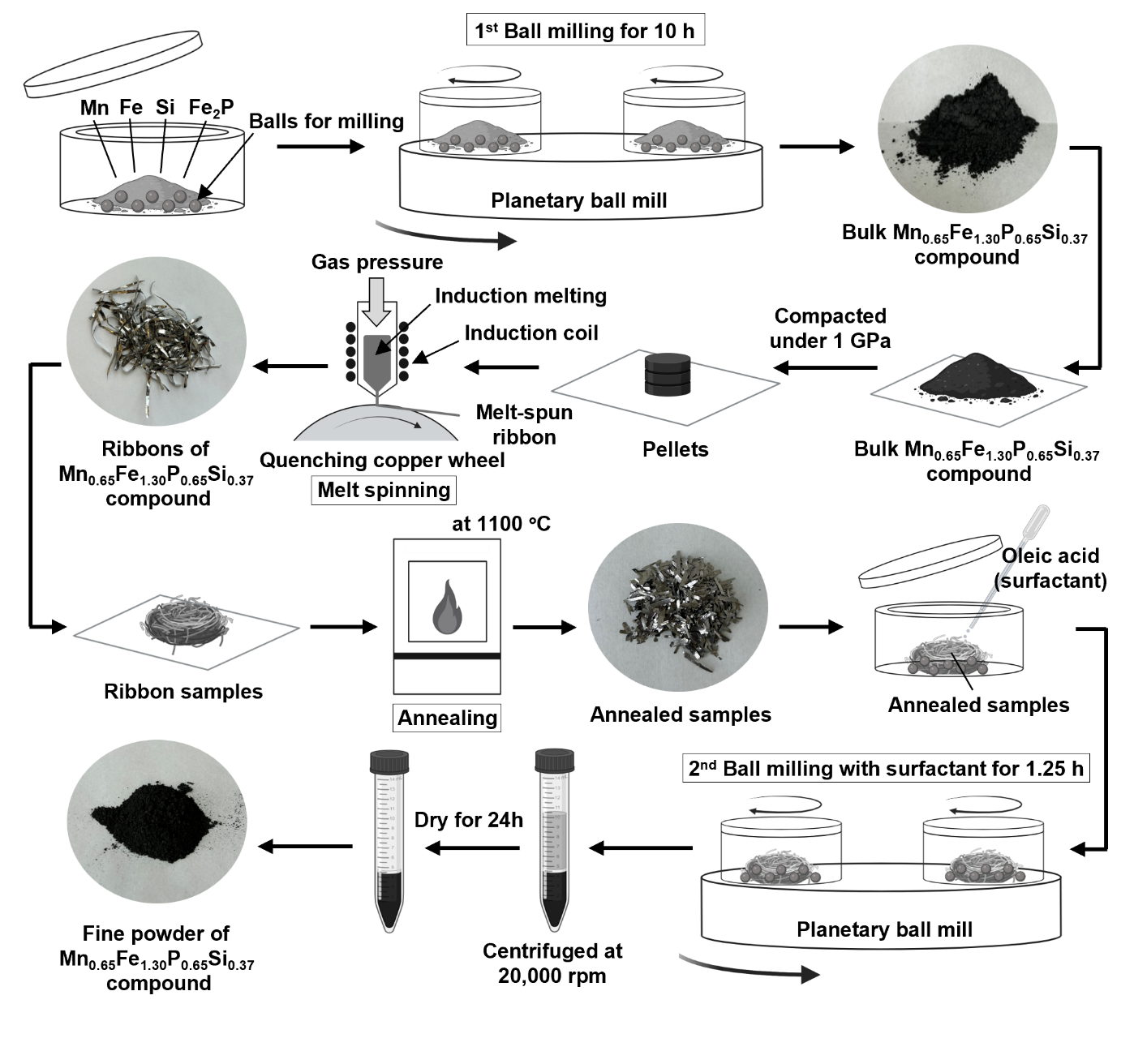


**Figure S1.** A schematic illustration of the fabrication of Mn_0.65_Fe_1.30_P_0.65_Si_0.37_ magnetocaloric material (MCM). Created in [BioRender.com](https://BioRender.com/mnp7u8t).


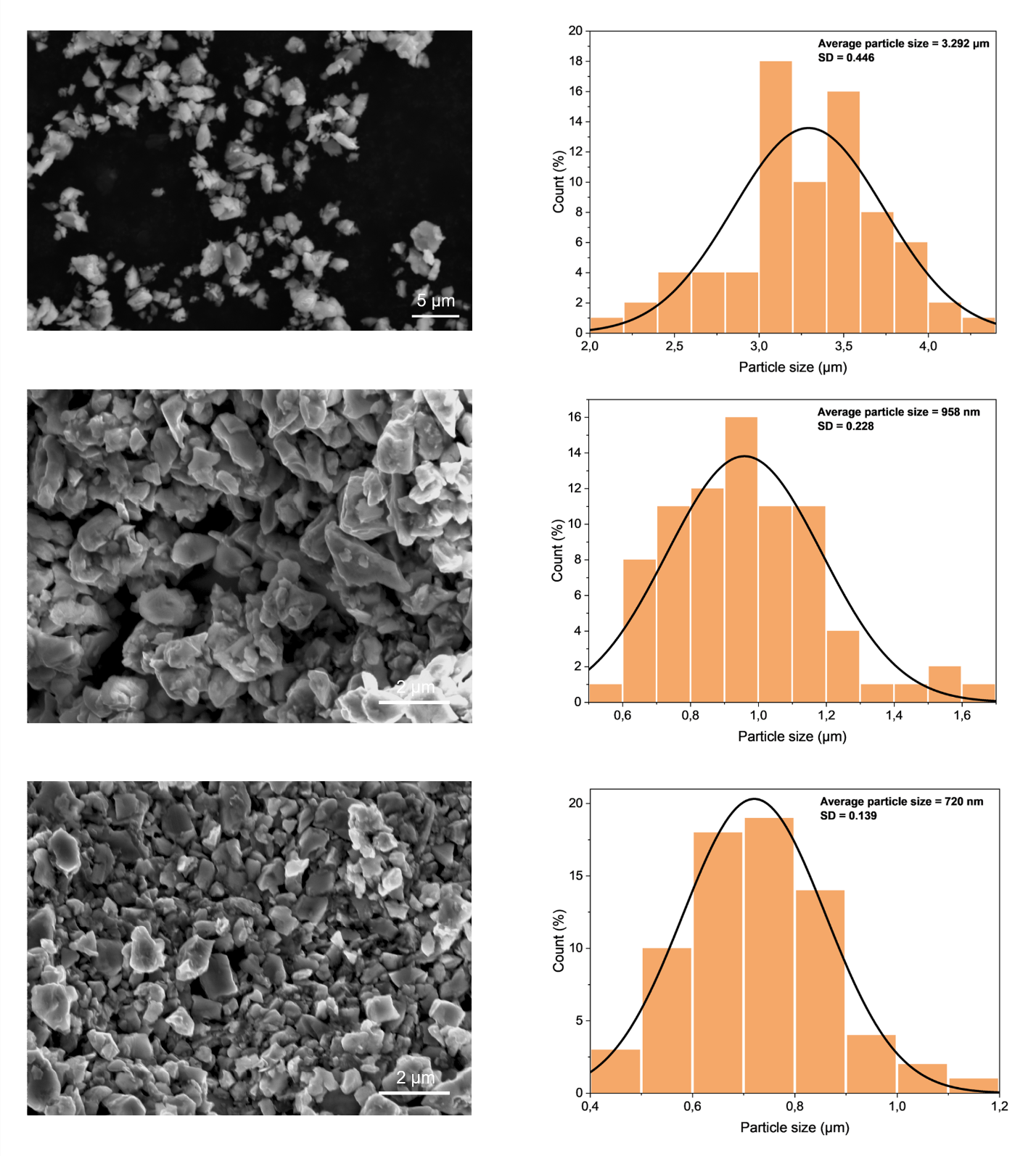


**Figure S2.** SEM images of Mn_0.65_Fe_1.30_P_0.65_Si_0.37_ polycrystalline particles obtained from different second ball-milling (BM) times.


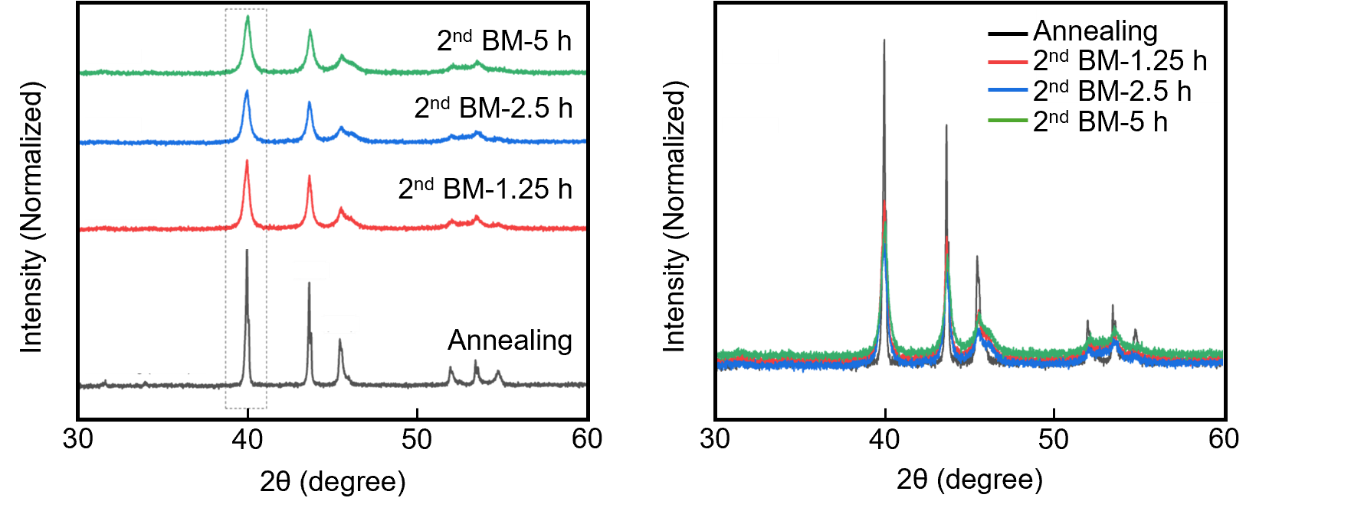


**Figure S3.** XRD patterns of Mn_0.65_Fe_1.30_P_0.65_Si_0.37_ (MCM) samples obtained from different ball-milling (BM) times.

**Table S1.** A summary of the properties measured in the Mn_0.65_Fe_1.30_P_0.65_Si_0.37_ sample.

| **1^st^ batch: Mn_0.65_Fe_1.30_P_0.65_Si_0.37_ sample** | | | | | |
| --- | --- | --- | --- | --- | --- |
| **Fabrication step** | **M_s_ at 5 K**  **(Am^2^ kg^-1^)** | **Tc**  **(K)** | **Tc _(Cooling)_**  **(K)** | **Tc _(Heating)_**  **(K)** | $\boldsymbol{\Delta}$**T_hys_**  **(K)** |
| Melt spinning | 103 | - | - | - | - |
| Annealing | 149 | 316 | 311 | 316 | 5 |
| 2^nd^ ball milling 1.25 h | 138 | 317 | 315 | 317 | 2 |

**Table S2.** A summary of the properties measured in the resynthesized Mn_0.65_Fe_1.30_P_0.65_Si_0.37_ sample.

| **2^nd^ batch: (resynthesized) Mn_0.65_Fe_1.30_P_0.65_Si_0.37_ sample** | | | | | |
| --- | --- | --- | --- | --- | --- |
| **2^nd^ Ball milling (BM) time (h)** | **M_s_ at 5 K**  **(Am^2^ kg^-1^)** | **Tc**  **(K)** | **Tc _(Cooling)_**  **(K)** | **Tc _(Heating)_**  **(K)** | $\boldsymbol{\Delta}$**T_hys_**  **(K)** |
| Annealing | 146 | 317 | 314 | 317 | 4 |
| 1.25 | 116 | 316 | 314 | 316 | 2 |
| 2.5 | 82 | 316 | 314 | 316 | 2 |
| 5 | 77 | 316 | 314 | 316 | 2 |


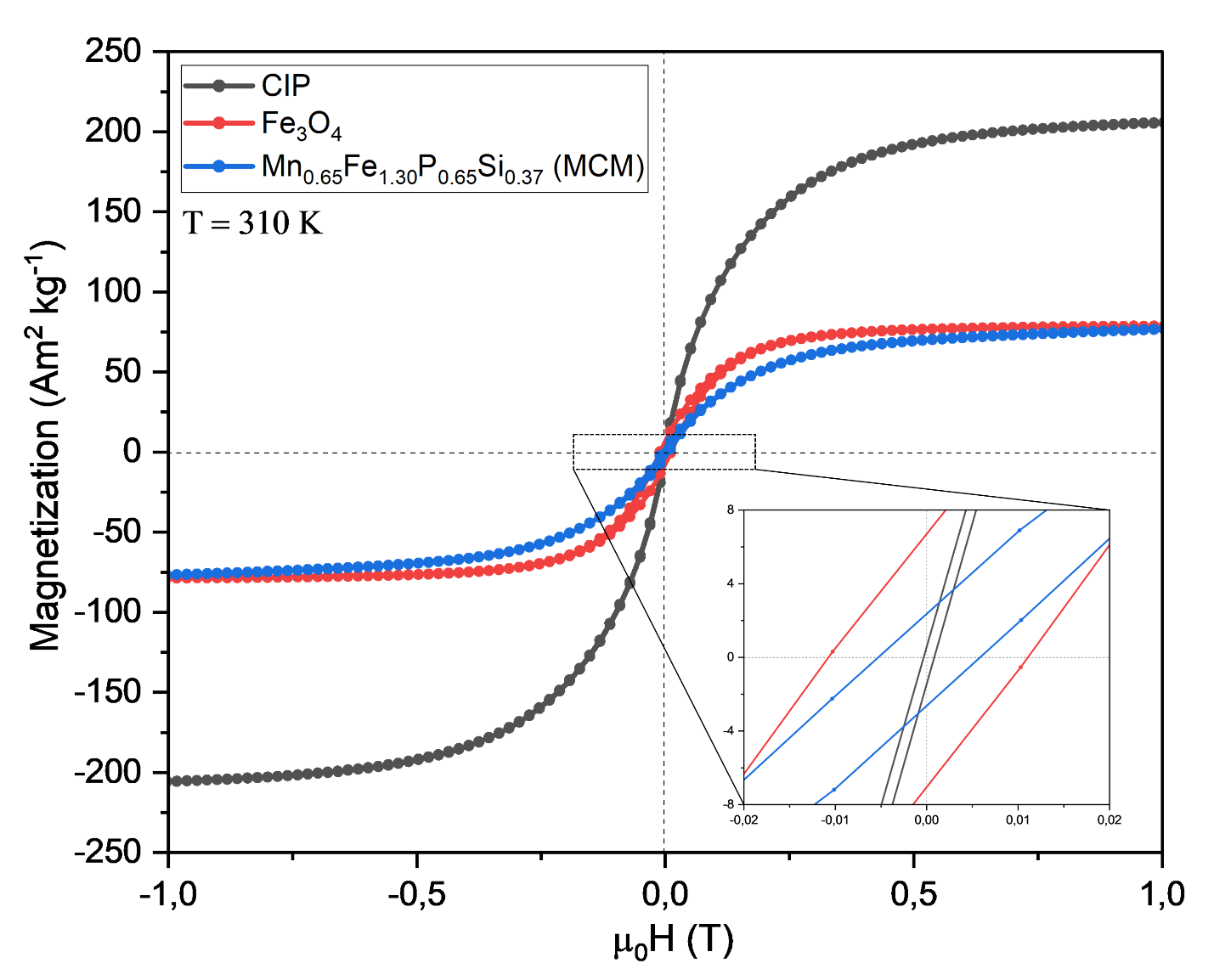


**Figure S4.** Magnetic hysteresis of CIP, Fe_3_O_4_, and Mn_0.65_Fe_1.30_P_0.65_Si_0.37_ magnetocaloric material (MCM) particle at 310 K.


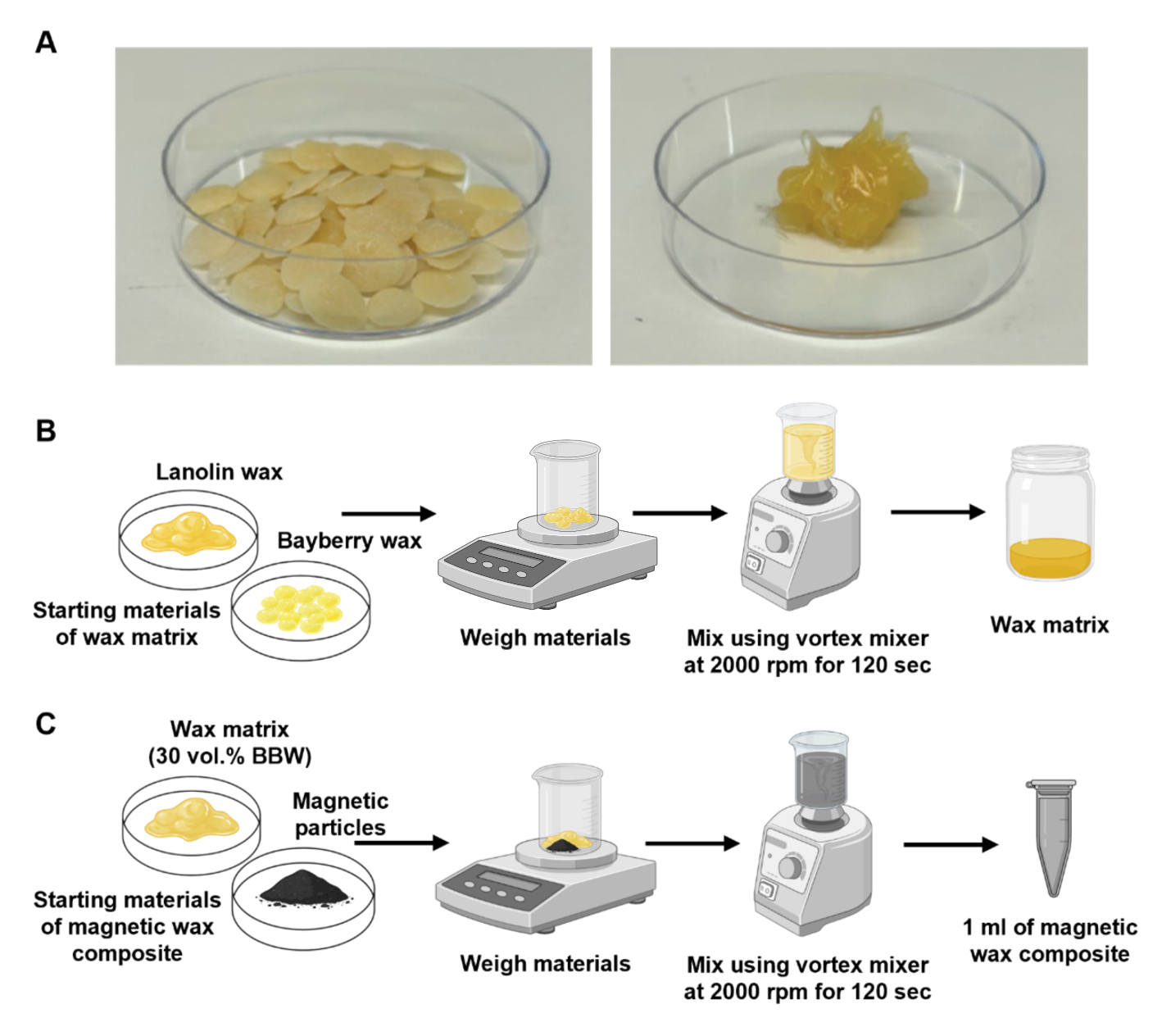


**Figure S5.** **A)** Two types of wax materials were employed to fabricate the transient membrane. Bayberry wax (left) is presented in the form of solid pellets, and lanolin wax (right) is characterized by its stickier and softer consistency. Schematic illustration of the fabrication of **B)** wax matrix and **C)** magnetic wax composites, respectively. Created in [BioRender.com](https://BioRender.com/k3b7vl1).


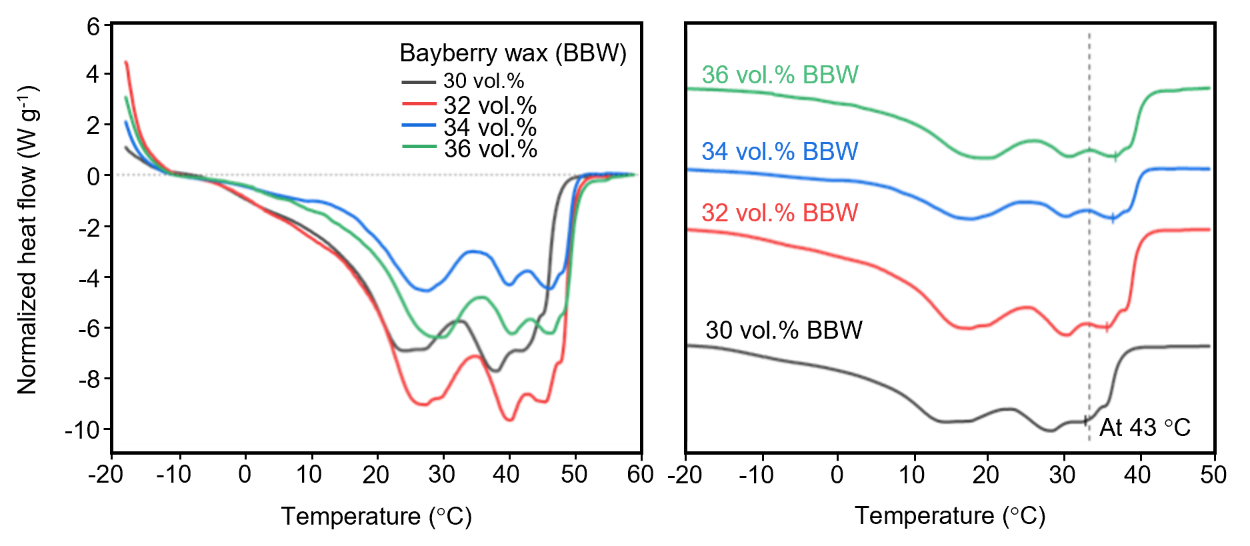


**Figure S6.** Thermal properties of wax matrices (bayberry wax mixed with lanolin wax). The second heat cycle of all wax matrices was obtained from DSC measurements. The normalized heat flow was investigated over the temperature range dependence of the bayberry wax (BBW) volume percentage, starting from 30 to 36 vol.%.

**Table S3.** Parameters used for calculation of specific loss power (*SLP*) values based on the temperature variations observed in Mn_0.65_Fe_1.30_P_0.65_Si_0.37_ (MCM) wax samples when subjected to different magnetic field strengths (6-10 mT) at 244 kHz. Values are presented with standard deviation {-}.

| **MCM sample with a 15 vol.% particle fraction** | | | | | |
| --- | --- | --- | --- | --- | --- |
| **Magnetic field, *µ_0_H***  **(T)** | **Heating rate,**  ***dT/dt* (°C s^-1^)** | **Particle mass, *m_Ps_* (g)** | ***SLP***  **(W kg^-1^)** | **Sample size** |  |
| 6 | 0.016 | 1.048 | 65 {2.54} | 2 |  |
| 7 | 0.027 |  | 108 {1.69} | 2 |  |
| 8 | 0.039 |  | 155 {7.91} | 2 |  |
| 9 | 0.060 |  | 241 {1.80} | 3 |  |
| 10 | 0.074 |  | 297 {12.39} | 3 |  |


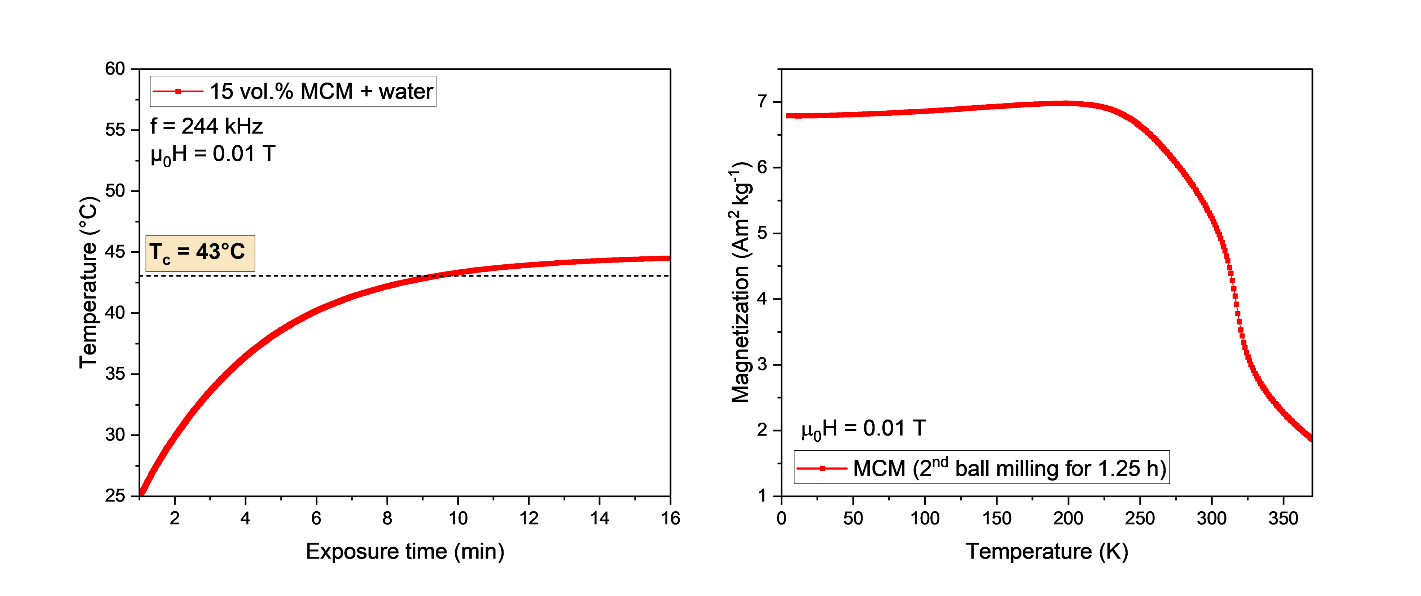


**Figure S7.** Thermal behavior of the Mn_0.65_Fe_1.30_P_0.65_Si_0.37_ sample under 0.01 T at 244 kHz (left). Temperature-dependent magnetization (*M-T*) curves of the Mn_0.65_Fe_1.30_P_0.65_Si_0.37_ sample obtained after the 2^nd^ ball milling for 1.25 h, measured at a magnetic field of 0.01 Tesla (T) (right).


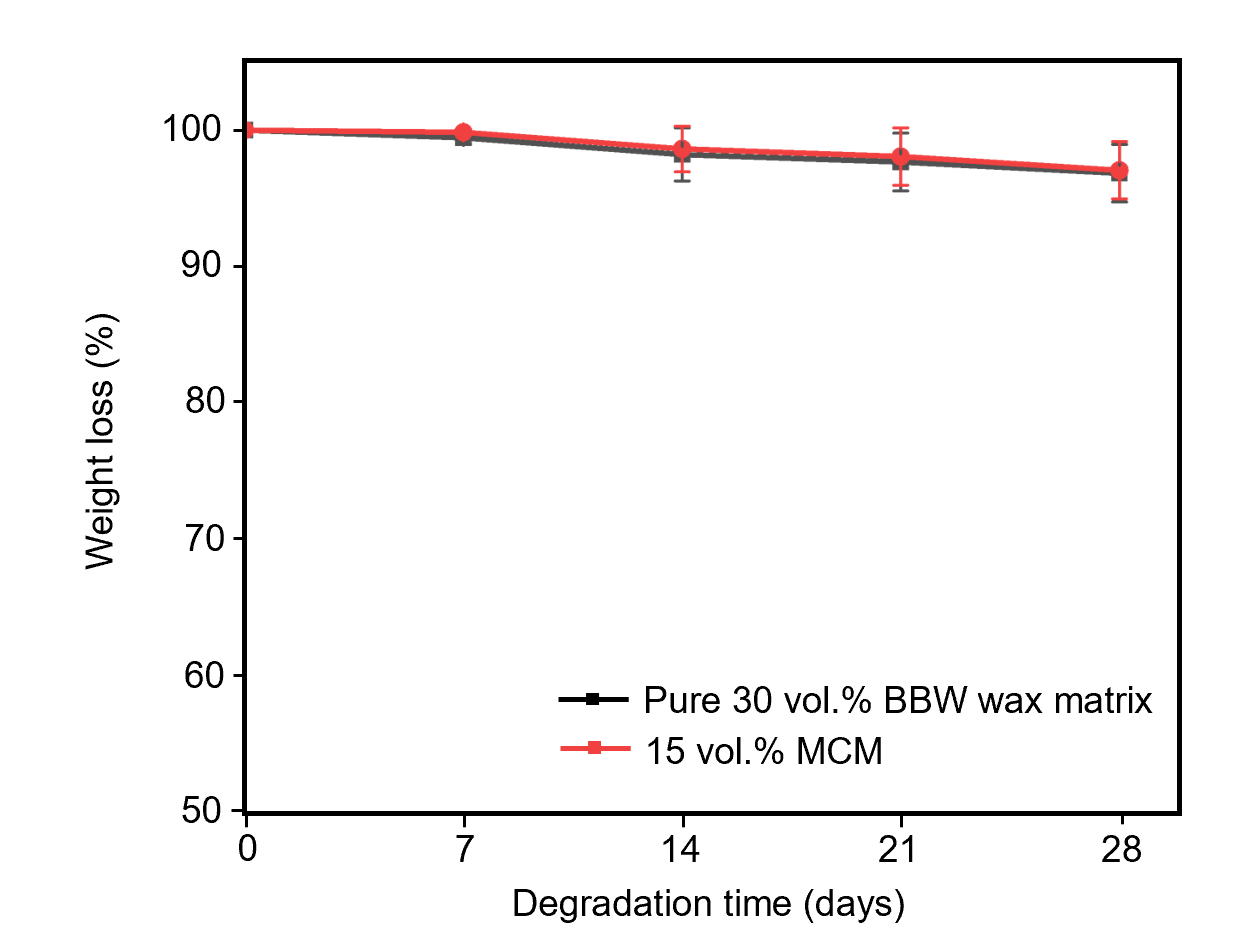


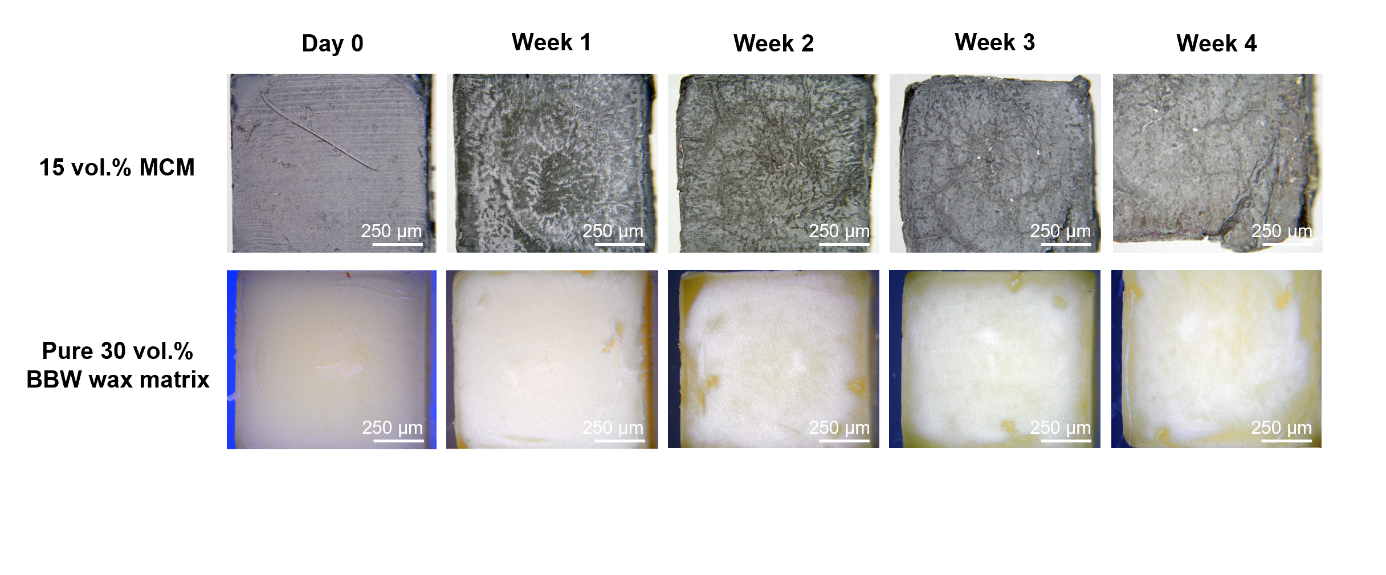


**Figure S8.** Degradation weight loss percentage (%) of the 15 vol.% MCM–wax composite and pure wax matrix after immersion in PBS (pH 7.4, 37 °C), along with corresponding morphological changes observed over 4 weeks.
